# Integrated multi-omics elucidates *PRNP* knockdown-mediated chemosensitization to gemcitabine in pancreatic ductal adenocarcinoma

**DOI:** 10.3389/fimmu.2025.1667835

**Published:** 2025-11-27

**Authors:** Bing Qi, Yuwei Wu, Ning Wang, Liumeng Duan, Yangbo Wei, Xuanrui Zhang, Jing Chen

**Affiliations:** 1 College of Life Sciences, North China University of Science and Technology, Tangshan, Hebei, China; 2 College of Basic Medical Sciences, North China University of Science and Technology, Hebei, Tangshan, China; 3 College of Ji Tang, North China University of Science and Technology, Tangshan, Hebei, China

**Keywords:** scRNA sequencing analysis, pancreatic ductal adenocarcinoma, cancer-associated fibroblasts, PRNP, chemotherapy

## Abstract

**Introduction:**

Pancreatic ductal adenocarcinoma (PDAC) is a highly aggressive malignancy with an extremely poor prognosis. Gemcitabine (GEM), the standard first-line chemotherapeutic agent for PDAC, often fails due to the development of drug resistance. This study aims to systematically investigate the mechanisms underlying gemcitabine resistance in PDAC and identify novel therapeutic targets.

**Methods:**

We integrated multi-omics data, including microarray, transcriptomic, proteomic, single-cell RNA sequencing, and spatial transcriptomic datasets. Machine learning algorithms were employed to screen for key genes associated with resistance. The correlation between candidate genes and drug-resistant phenotypes was inferred using pancreatic cancer cell lines, mouse models, and clinical patient data. Functional and mechanistic studies were subsequently conducted through in vitro cellular experiments.

**Results:**

Our findings identify the prion protein gene (*PRNP*) as a key gene associated with chemoresistance. *PRNP* expression is significantly elevated in PDAC patients treated with gemcitabine and correlates with the resistant phenotype. Cellular experiments confirmed that gemcitabine exposure upregulates PRNP expression, while *PRNP* knockdown significantly reduces the half-maximal inhibitory concentration of gemcitabine and enhances its cytotoxicity. Mechanistic studies demonstrate that *PRNP* drives resistance through dual pathways: it promotes epithelial-mesenchymal transition (EMT), enhancing cellular invasiveness, and suppresses ferroptosis by upregulating the expression of ferroptosis-related proteins SLC7A11 and GPX4, thereby maintaining redox homeostasis. Further single-cell and spatial transcriptomic analyses revealed that PRNP is predominantly enriched in a specific subset of cancer-associated fibroblasts (CAFs) following chemotherapy, which is associated with the establishment of an immunosuppressive tumor microenvironment.

**Discussion:**

This study demonstrates that *PRNP* is a key regulator of gemcitabine resistance in PDAC, modulating EMT, ferroptosis, and the tumor immune microenvironment. Targeting *PRNP* represents a promising therapeutic strategy to reverse gemcitabine resistance and may hold significant potential for clinical translation in PDAC treatment.

## 1 Introduction

Pancreatic cancer (PC) is a malignant tumor originating from pancreatic tissues of the digestive system. Based on its cellular origin, pancreatic cancer can be classified into several subtypes, including pancreatic endocrine tumors, intraductal papillary mucinous neoplasms, adenocarcinomas, and metastatic pancreatic cancers (1, 2). Among these, PDAC is the most common subtype, arising from the epithelial cells lining the pancreatic ducts and exhibiting a strong propensity for invasion and metastasis (3). The early stages of PDAC are often asymptomatic, making timely detection challenging and leading to delayed diagnosis and subsequent difficulties in treatment (4).

In recent years, a variety of novel therapeutic approaches for pancreatic cancer have been developed, including chemotherapy, radiotherapy, surgical resection, and immunotherapy. Despite these advances, gemcitabine remains a cornerstone of pancreatic cancer treatment and is widely used as a first-line chemotherapeutic agent (5). Gemcitabine exerts its antitumor effects through multiple mechanisms, primarily by inhibiting DNA synthesis and cell division. In clinical practice, it can be administered as a monotherapy or in combination with other chemotherapeutic drugs, yielding favorable synergistic effects (6). However, a major clinical challenge is the development of chemoresistance in pancreatic cancer patients during gemcitabine treatment, and the underlying mechanisms of this resistance remain incompletely understood.

Studies have demonstrated that the prion gene family comprises four members within cells: *PRNP* (PrPC), *PRND* (Doppel), *PRNT* (PRT), and *SPRN* (Shadoo) (7). Among these, *PRNP* which encodes a copper homeostasis-associated prion protein—has attracted extensive research attention. *PRNP* is predominantly expressed in the central nervous system but is also detected in digestive organs, including the gastrointestinal tract and pancreas (8). Previous studies have linked *PRNP* overexpression to the initiation and progression of multiple cancers, such as gastric, colorectal, lung, and breast cancers, as well as pancreatic cancer and gliomas. This overexpression is closely associated with poor prognosis, dysregulated cell proliferation, enhanced invasion and metastasis, and drug resistance in cancer patients (9–12). Additionally, *PRNP* is primarily expressed in Schwann cells and axons of the peripheral nervous system (13). During cancer chemotherapy, upregulation of *PRNP* in cancer cells can induce EMT, thereby promoting the transdifferentiation of these cells into CAF-like phenotypes (14–16). Furthermore, interactions between CAFs and Schwann cells may facilitate tumor neural invasion, potentially through the regulation of *PRNP* expression (17).

In the present study, we aimed to investigate the mechanism by which *PRNP* modulates the response of pancreatic cancer to gemcitabine treatment. To achieve this goal, we integrated multi-omics data and conducted *in vitro* cellular experiments to identify novel therapeutic targets and strategies for pancreatic cancer. Our findings provide new insights for the precise, targeted diagnosis and treatment of pancreatic cancer patients.

## 2 Materials and methods

### 2.1 Data download and processing

Single-cell RNA sequencing (scRNA-seq) datasets of pancreatic cancer were downloaded from the Gene Expression Omnibus (GEO) database under the accession numbers GSE186960, GSE189753, GSE222952, and GSE205013. These datasets were processed using the Seurat package. High-quality cells were meticulously filtered on the basis of criteria including nFeature > 500, min.cells > 3, and mitochondrial gene expression < 20%. Subsequent data normalization was performed, and the top 2,000 most variable genes were selected for further analysis. To standardize expression levels and mitigate batch effects, the ScaleData function and Harmony algorithm were applied. Classical marker genes were used to annotate distinct cell populations within the dataset. Differential genes were identified using the following criteria: adjusted P value < 0.05, absolute average log2-fold change > 1, and a significant percentage difference between subclusters > 0.1.

Microarray and transcriptome datasets from pancreatic cancer cell lines, bearing the following accession numbers, were harnessed: GSE105083, GSE106336, GSE140077, GSE152121, GSE152123, GSE153460, GSE172303, GSE223303, GSE228106, GSE249302, GSE35141, GSE78982, GSE79953, GSE80617, and GSE97766. To guarantee precise gene evaluation, these bulk datasets were analyzed individually rather than collectively. For the RNA-seq data, specifically SRP303224, quality control was rigorously conducted via FastQC. Read trimming was then executed with Fastp, followed by alignment to the GRCh38 reference genome using HISAT2. Finally, read counting was performed using FeatureCounts. Differential genes in bulk RNA-seq data were identified using the criteria: adjusted P value < 0.05 and absolute log_2_-fold change > 0.5.

Using data from The Cancer Genome Atlas (TCGA) on pancreatic cancer, patients were categorized into high-PRNP expression and low-PRNP expression groups on the basis of the median expression level of the *PRNP* to explore its role in expression. To deduce stromal cell type scores within both the high- and low-expression patient groups, we employed the EPIC, xCell, MCPcounter and estimate packages.

The single-cell spatial transcriptomics dataset, with the accession number GSE235452, was processed via the Seurat package. Data normalization and batch effect correction were executed via the SCTransform function. Clustering was then conducted via the FindNeighbors and FindClusters functions, culminating in cell type scoring, which was accomplished via the AddModuleScore function.

Proteomic data were retrieved from the ProteomeXchange database (proteomexchange.org) under the accession number PXD030861. For proteomic identification and quantification, MaxQuant (version 2.4.2) was used with default settings, referencing the human Swiss-Prot protein database.

### 2.2 Monocle trajectory analysis

Pseudotime trajectories for single cells were analyzed via the Monocle2 package. The functions newCellDataSet, estimateSizeFactors, and estimateDispersions were leveraged to carry out these analyses. The detectGenes function was utilized to filter out low-quality cells, applying a min_expr threshold of 0.1.

### 2.3 Protein network interaction and pathway enrichment

The protein–protein interaction (PPI) network for *PRNP* was constructed via the GeneMANIA database, complemented by enrichment analysis. To delve into the molecules associated with *PRNP*, the ClusterProfiler package was used for comprehensive Gene Ontology (GO), Kyoto Encyclopedia of Genes and Genomes (KEGG), and Gene Set Enrichment Analysis (GSEA). Furthermore, Gene Set Variation Analysis (GSVA) was implemented through the dedicated GSVA package, while visualizations were refined via the Gseavis package for enhanced clarity.

### 2.4 Machine learning filtering genes

Three machine learning algorithms LASSO, SVM-RFE and RF were employed to explore disease states. The LASSO algorithm was utilized for variable selection and complexity regularization. SVM-RFE was implemented to identify the most relevant key genes through recursive feature elimination. For the RF algorithm, its principle involved deriving reliable results from a large ensemble of underlying tree models. Finally, the optimal key genes were determined by intersecting the common features identified across all three algorithms. This integrative approach ensured the selection of robust biomarkers with cross-method validation. The predictive value of core genes is assessed by ROC. This allows the prediction of core genes as biomarkers as well as diagnostic capabilities.

The diagnostic potential of candidate genes in pancreatic adenocarcinoma was validated using the GEPIA2 platform (http://gepia2.cancer-pku.cn/), which integrates standardized RNA-seq data from TCGA-PAAD (179 tumors) and GTEx-Pancreas (171 normals).

### 2.5 Drug sensitivity analysis of high PRNP group and low PRNP group

Gemcitabine sensitivity in pancreatic cancer was investigated by leveraging the Genomics of Drug Sensitivity in Cancer (GDSC) database to estimate individual patient responses. The R package oncoPredict was subsequently used to calculate Gemcitabine sensitivity scores. Concurrently, the Tumor Immune Dysfunction and Exclusion (TIDE) algorithm was applied to predict immunotherapy response and evaluate immune escape potential.

### 2.6 Cell culture and reagents

Gemcitabin (Macklin) has a purity of > 95.0%. The human pancreatic cancer cell lines PANC-1 and ASPC-1 were kindly provided by Professor Yongsu Zhen. The cell lines were maintained in a 37 °C incubator under 5% CO_2_ conditions. While ASPC-1 cells thrived in RPMI-1640 medium supplemented with 10% FBS, PANC-1 cells exhibited optimal growth in DMEM under the same serum conditions. The cells were seeded at a density of 5×10^5^ in 6-well dishes and transfected with 100 pmol specific Small interfering RNAs (siRNAs) for 48h in the presence of 4 μL Lipo8000 (Beyotime) transfection reagent. siRNAs were purchased from GenePharma. The following antibodies were used: E-cadherin(Huabio); Slug(Wanleibio); Snail(Wanleibio); PRNP(UpingBio); P53(Huabio); BAX(Huabio); Bcl2(Huabio); β-actin(ABclonal); SLC7A11(Biodragon); GAPDH(ABclonal); GPX4(Huabio). Ferroptosis inhibitors were purchased from Cayman.

### 2.7 Quantitative reverse-transcription polymerase chain reaction

Total RNA was isolated using the Zsgentech 6-min Rapid RNA Extraction Kit and reverse-transcribed to cDNA. Relative mRNA quantification was performed via the 2^-ΔΔCt^ method with GAPDH as the endogenous control. The primers used are shown in **Table 1**. The siRNA sequences are shown in **Table 2**.

**Table 1:** Primer sequences for qRT–PCR.

Gene	Sequences
*PRNP*	F: 5’-ACAACTTTGTGCACGACTGC-3’ R: 5’-TGGAGAGGAGAAGAGGACCA-3’
*GAPDH*	F: 5’-CACCCACTCCTCCACCTTTGAC -3’ R: 5’-GTCCACCACCCTGTTGCTGTAG -3’

**Table 2:** siRNA sequences used for transfection.

siRNA	Sequences
NC	F:5’-UUCUCCGAACGUGUCACGUTT-3’ R:5’-ACGUGACACGUUCGGAGAATT-3’
siRNA-1115	F:5’- GAUCGAGCAUGGUCCUCUUTT-3’ R:5’- AAGAGGACCAUGCUCGAUCTT -3’
siRNA-449	F:5’- GGAUGCUGGUUCUCUUUGUTT-3’ R:5’- ACAAAGAGAACCAGCAUCCTT -3’
siRNA-1031	F:5’- CCGACGUUAAGAUGAUGGATT -3’ R:5’- UCCAUCAUCUUAACGUCGGTT-3’

### 2.8 MTT assays of cell proliferation

Pancreatic cancer cells, ranging from 3000 to 4000 per well, were seeded in 96-well plates and cultured for 24 hours. They were then treated with GEM in serum-free media for 24–48 hours. Following treatment, MTT was added, and the cells were incubated for an additional 4 hours. DMSO was subsequently added to dissolve the formed crystals, and the absorbance was measured at 570 nm. The IC50 values were calculated via GraphPad 9.0.

### 2.9 Trypan blue and clone survival experiments

Cell viability was evaluated via the trypan blue exclusion method. The cells were plated in 6-well plates at a density of 5×10^5^ cells per well in serum-free medium supplemented with GEM and incubated at 37°C with 5% CO_2_ for 48 hours. All the cells from each well were subsequently centrifuged into a tube and stained with a 0.5% trypan blue solution for 3 minutes. The stained cells were observed under an inverted microscope, and viability was determined by counting the viable cells.

To assess the cell proliferation capacity via the clonogenic survival assay, pretreated cells were plated in 6-well plates. Once adhered, the cells were categorized into six groups on the basis of experimental requirements: Control (C), GEM (G), siPRNP (SI), siPRNP+GEM (SI+G), Ferrostatin-1(Fer-1) and siPRNP+Ferrostatin-1(SI+Fer-1). Each group included at least three replicate wells. The cells were incubated until most of the cell clusters comprised approximately 50 cells. The samples were then fixed with paraformaldehyde and stained with crystal violet for 20 minutes. The formed cell colonies were observed under an inverted microscope.

### 2.10 Cell scratch and invasion migration assays

Cells in optimal growth conditions were seeded into 6-well plates. Upon adherence, a scratch was introduced into the monolayer via a 100 µl pipette tip. Following a rinse with PBS, the cells were treated according to the experimental protocol, with each group featuring at least three replicate wells. Culture medium was added, and the scratch area was monitored at various time points via an inverted microscope.

For the invasion assay, 100 µL of Matrigel was added to the chamber. A complete medium containing 20% FBS was placed in the lower chamber of a 24-well plates, and 100 µL of the treated cell suspension was evenly distributed into the upper chamber. After the specified incubation period, the medium was discarded, and the cells were gently rinsed twice with PBS. The cells were subsequently fixed with 4% paraformaldehyde for 10 minutes and stained with crystal violet for another 10 minutes. Nonmigrated cells on the upper surface were carefully removed with a wet cotton swab, and the cells were observed and photographed under an inverted microscope.

For the migration assay, a similar procedure was used. Specifically, 500 µL of complete medium containing 20% FBS was added to the lower chamber of a 24-well plates, and 100 µL of the treated cell suspension was evenly placed into the upper chamber. The subsequent steps mirrored those of the Transwell invasion assay.

### 2.11 Mitochondrial function assays

JC-1 solution was added to the 6-well plates at a concentration of 10 μM, followed by incubation in the dark at 37°C for 15 minutes. During this period, the plate was gently shaken every 4 min to prevent dye agglomeration. After incubation, the dye was aspirated, and the cells were thoroughly washed twice with PBS to eliminate any unbound JC-1 dye. The cells were then examined and photographed under an inverted fluorescence microscope.

For Reactive Oxygen Species (ROS) detection, the instructions provided by the ROS detection kit from Report Biotech were followed meticulously. Specifically, DCFH-DA reagent (10 mM) was diluted 1000 times with serum-free medium to prepare the working solution. The supernatant from the 6-well plates was aspirated and discarded, and 1 mL of the DCFH-DA working solution was added to each well. After staining, the supernatant was removed, and the cells were rinsed three times with serum-free cell culture medium to ensure the complete removal of unbound DCFH-DA. The cells were subsequently observed and photographed under an inverted fluorescence microscope. Additionally, flow cytometry was employed to quantify the fluorescence intensity before and after transfection or drug stimulation by selecting the appropriate fluorescence channel, with an excitation wavelength of 488 nm and an emission wavelength of 525 nm.

### 2.12 Glutathione content test

Pancreatic cancer cells in the logarithmic growth phase were plated into 6-well plates. Upon adherence and reaching a growth density exceeding 80%, the medium was exchanged with medium containing the drug for a 48-hour treatment period. Following treatment, the cells were harvested, and detection reagents, prepared according to the instructions of the GSH level detection kit, were added. The absorbance of the samples at a wavelength of 412 nm was then measured via a microplate reader. The relative GSH levels of each group were subsequently calculated, statistically analyzed, and graphically represented.

### 2.13 MDA detection

Pancreatic cancer cells were lysed in an ice bath environment, and the control solution, standard and sample were added to the centrifuge tube according to the standard and working solution configured in the MDA kit (Boxbio) instructions, and then the working solution was added. After mixing, heat at 100 °C for 15 mins. After cooling to room temperature, centrifuge, the supernatant was added to a 96-well plates, and the absorbance of each well (532 nm) was measured by a microplate reader to calculate the MDA concentration.

### 2.14 AO/EB double fluorescence staining was used to detect the degree of apoptosis

Acridine orange (AO) and ethidium bromide (EB) were combined in a 1:1 ratio to create the working solution. This solution was dispensed into each well, and the cells were subsequently incubated at 37°C for 15 minutes. Following incubation, alterations in nuclear morphology were observed under a fluorescence microscope.

### 2.15 Western blot

Pancreatic cancer cells with good growth status were inoculated into 6-well plates, and after the cells were treated according to the experimental requirements, the cells were collected, the proteins in the cells were extracted, and then the protein expression was detected. The steps include: reagent preparation, protein sample preparation, determination of protein content, SDS-PAGE electrophoresis, membrane transfer, immunoreaction, chemical reflectance development and fixation, and gel image analysis.

### 2.16 Flow cytometry to detect apoptosis

Pancreatic cancer cells were cultivated to the logarithmic growth phase and subsequently treated with drugs for 48 hours, while a negative control was established. The cells were then washed twice with the prepared PBS buffer and resuspended in 1× Buffer to a density of 1×10^6^/mL. Using a flow cytometry kit, the resuspended cells were stained with 5 μL of each Annexin V-FITC solution and PI solution for 15 minutes at room temperature. After staining, the cells were washed with PBS buffer and analyzed for apoptosis via flow cytometry. The results were further analyzed with FlowJo software.

### 2.17 Statistical methods

In this study, we utilized the R language (version 4.4.0) for analysis. Each experiment was independently replicated three times or more, and the results are presented as the means ± standard deviations. A t test was used for comparisons between two groups, whereas one-way ANOVA was used for comparisons among multiple groups. The statistical analysis and plotting of group values were conducted via ImageJ and GraphPad Prism 9.0 software. *p* < 0.05 indicated that the difference was statistically significant.

## 3 Results

### 3.1 Gemcitabine stimulates the expression of *PRNP* in cell lines

**Figure 1:**
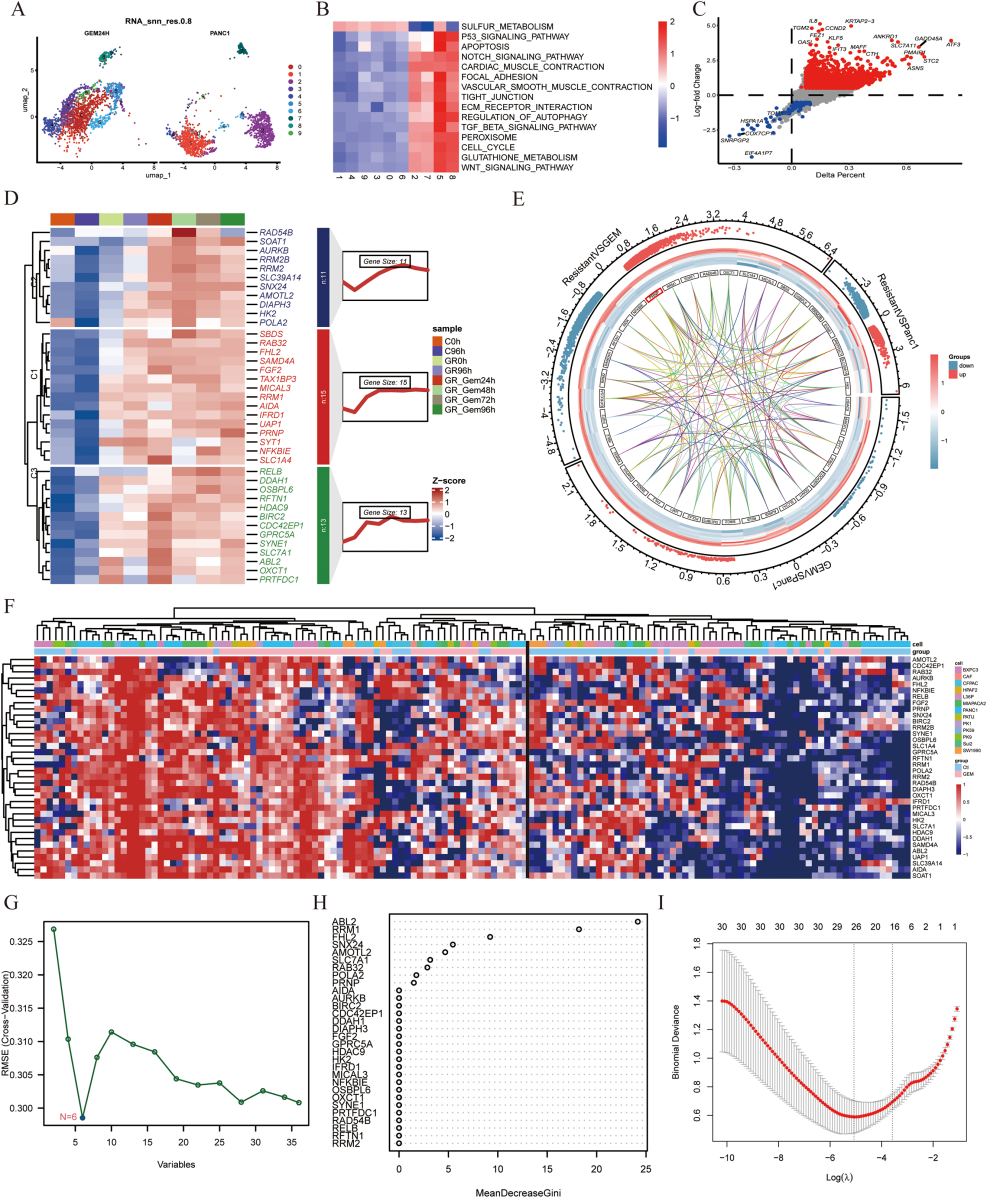
Expression of the *PRNP* gene in different cell lines treated with gemcitabine **(A)** UMAP plot of the PANC-1 cell line treated with gemcitabine. **(B)** Heatmap of GSVA pathway enrichment in subclusters. **(C)** Volcano plot of differential gene expression between subclusters 5 and 8 and subclusters 2 and 7. **(D)** Heatmap of the expression profiles of 39 proteins. **(E)** Chord diagram of transcriptome data from GSE80617 and GSE153460. **(F)** Heatmap of the expression of 39 genes in various cell lines. **(G)** Identifying biomarkers by SVM-RFE. **(H)** Identifying biomarkers by Random Forest algorithm. **(I)** Identifying biomarkers by LASSO algorithm.

The PANC-1 cell line is widely used in pancreatic cancer research; thus, we prioritized PANC-1 data for our investigations. By analyzing scRNA-seq data from PANC-1 cells treated with gemcitabine for 24 hours, we generated a uniform manifold approximation and projection (UMAP) plot to visualize cellular distribution (**Figure 1A**). At the single-cell resolution, we excluded PANC-1 cells that exhibited no response to gemcitabine. Through GSVA enrichment analysis, clusters 5 and 8 were associated with gemcitabine stimulation, highlighting the upregulation of signaling pathways, including the TGF-β, WNT, and ECM receptor pathways. This upregulation facilitated the fibrotic transformation of cancer cells, thereby contributing to gemcitabine resistance (**Figure 1B**). To delve deeper into the genetic alterations within these two subclusters, we conducted differential analysis between subclusters 2 and 7 from the control group and subclusters 5 and 8. Stringent criteria were applied—adjusted P value < 0.05, absolute average log2-fold change > 1, and a significant percentage difference between subclusters > 0.1. We identified 14 downregulated genes and 737 upregulated genes (**Figure 1C**). Furthermore, via proteomics technology, we identified 39 proteins that demonstrated remarkable stability in gemcitabine-resistant PANC-1 cells (**Figure 1D**).

Furthermore, to identify genes inherently resistant to gemcitabine-induced cell death, we integrated two gene expression datasets, GSE80617 and GSE153460, via the SVA package and conducted three rigorous rounds of differential analysis. By applying stringent criteria of an adjusted P value < 0.05 and an absolute log2-fold change > 0.5, we identified 39 genes that were upregulated upon drug stimulation and played pivotal roles in regulating the emergence of drug-resistant phenotypes (**Figure 1E**). These genes included *ABL2, AIDA, AMOTL2, AURKB, BIRC2, CDC42EP1, DDAH1, DIAPH3, FGF2, FHL2, GPRC5A, HDAC9, HK2, IFRD1, MICAL3, NFKBIE, OSBPL6, OXCT1, POLA2, PRNP, PRTFDC1, RAB32, RAD54B, RELB, RFTN1, RRM1, RRM2, RRM2B, SAMD4A, SBDS, SLC1A4, SLC39A14, SLC7A1, SNX24, SOAT1, SYNE1, SYT1, TAX1BP3* and *UAP1*.

To evaluate the adaptability of a gene set across diverse pancreatic cancer cell lines and effectively filter out false positives, transcriptome and microarray datasets encompassing both GEM-treated groups and GEM-resistant pancreatic cancer cell lines were meticulously selected. The expression data for the 39 genes were extracted from each dataset, normalized, and subsequently integrated into a heatmap for comprehensive visualization (**Figure 1F**).

**Figure 2:**
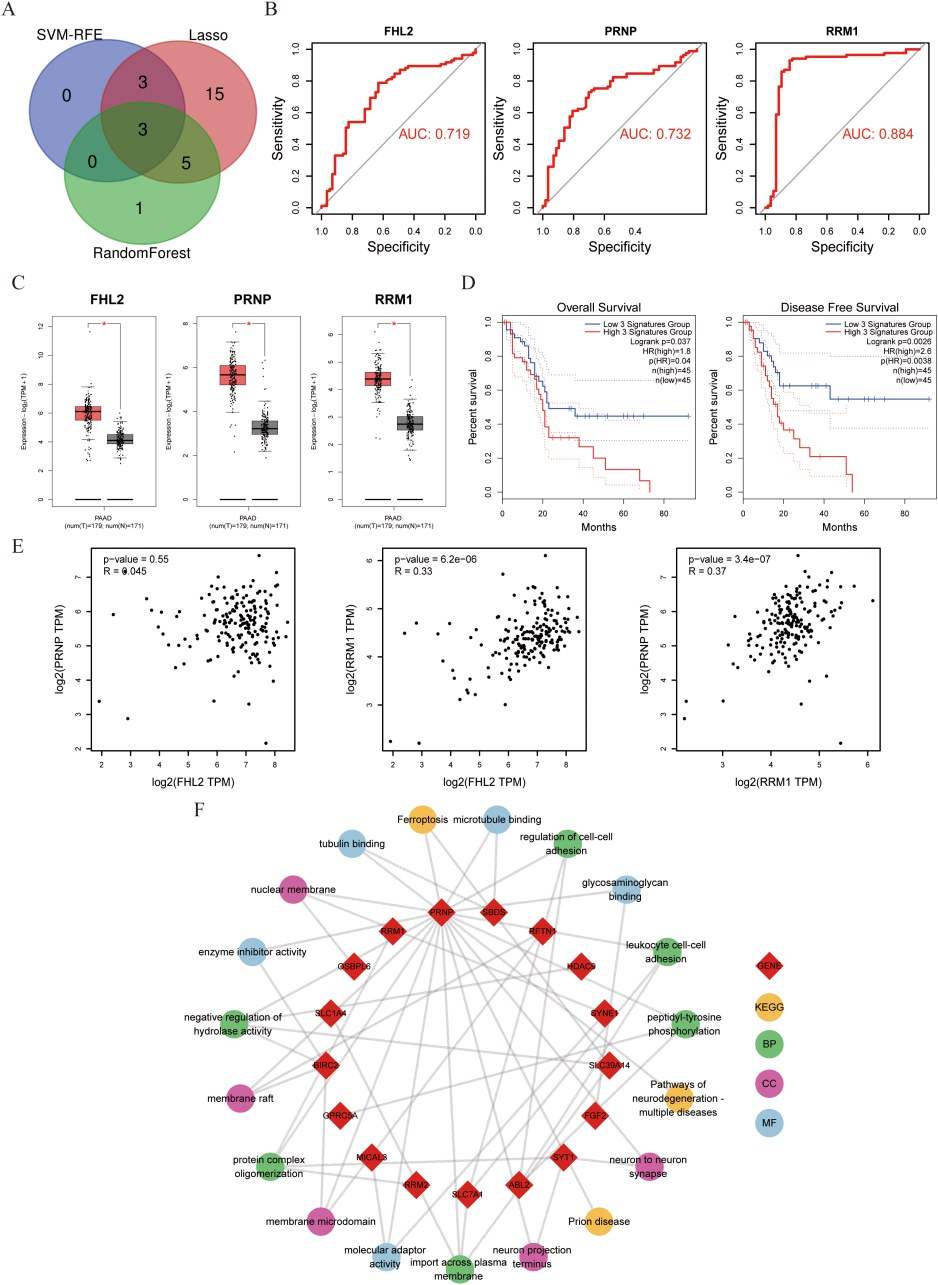
Evaluation of *FHL2*, *PRNP*, and *RRM1* genes with pathway enrichment **(A)** Venn diagram displaying three genes intersected by machine learning algorithms. **(B)** Diagnostic effectiveness of *FHL2*、*PRNP* and *RRM1* in ROC curves. **(C)** Expression of *FHL2*、*PRNP* and *RRM1* in PAAD patients compared to controls. **(D)** Three genes OS and DFS Survival curves about PAAD. **(E)** Scatter plot of correlation between the three genes. **(F)** Chord diagram of *PRNP* gene pathway interactions.

To systematically identify clinically relevant genes associated with gemcitabine response in pancreatic cancer, we implemented a multi-algorithm integration approach starting with 39 candidate genes. The LASSO regression model identified 26 prognostic genes, while the random forest algorithm selected 9 key features. Support vector machine recursive feature elimination (SVM-RFE) refined the list to 6 core genes (**Figures 1G–I**). Venn diagram analysis of the three algorithm outputs revealed *FHL2*, *PRNP*, and *RRM* as consistently selected candidates (**Figure 2A**). These genes exhibited predictive value for gemcitabine sensitivity, with ROC analysis showing area under the curve (AUC) values > 0.7 (**Figure 2B**). Validation using the TCGA-PAAD cohort (n=178) confirmed significant tumor-specific overexpression compared to normal controls (*p* < 0.05) (**Figure 2C**). Survival analysis revealed that elevated expression of all three genes correlated with poorer overall survival (OS) and disease-free survival (DFS) (*p* < 0.05) (**Figure 2D**). Notably, *PRNP* showed the strongest positive correlation with RRM1 (Pearson’s R = 0.37, *p* < 0.05), supporting its prioritization as a potential therapeutic target for overcoming gemcitabine resistance in pancreatic cancer (**Figure 2E**).

Next, KEGG and GO enrichment analyses were performed on the selected gene set to pinpoint *PRNP*-associated genes and signaling pathways. The results revealed that the *PRNP* is intricately linked with multiple pivotal pathways, including ferroptosis, neurodegenerative diseases, actin filament binding, and microtubule binding signaling pathways. These findings underscore the strong correlation between *PRNP* expression and vital biological processes such as cell proliferation, cell migration, and cell apoptosis (**Figure 2F**).

### 3.2 The role of *PRNP* in tumor immune microenvironment and drug sensitivity

Using transcriptome data from the TCGA-PAAD cohort, we stratified patients into high-PRNP and low-PRNP expression groups based on the median *PRNP* expression level. This stratification allowed us to systematically analyze the relationship between *PRNP* expression and both drug sensitivity and immune cell infiltration.

**Figure 3:**
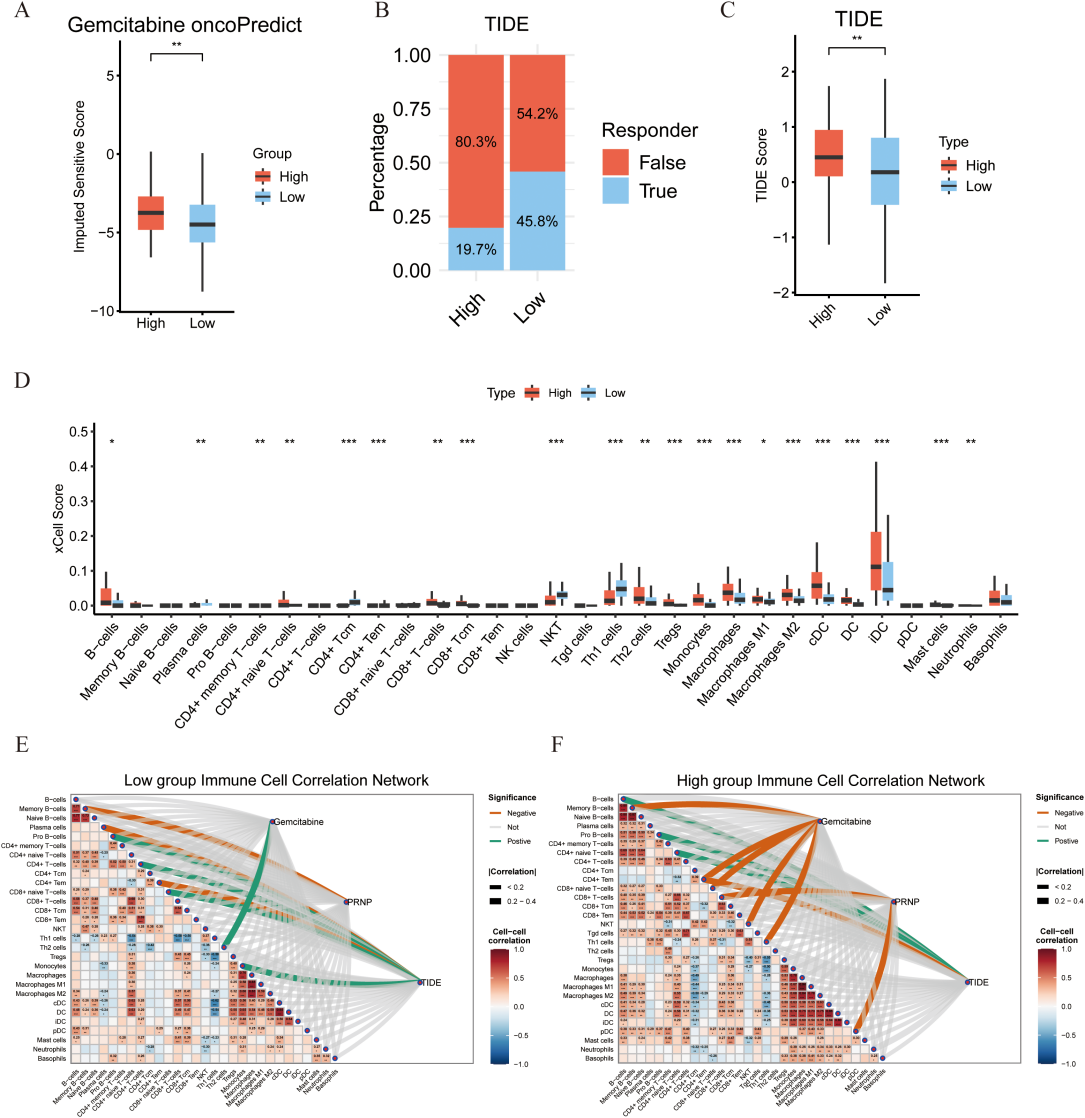
Associations between *PRNP* gene expression, sensitivity to immune checkpoint therapy, and characteristics of the immune microenvironment. **(A)** Differences in gemcitabine sensitivity between high and low *PRNP* gene expression groups. **(B)** Proportion of predicted responses to immunotherapy in high and low PRNP gene expression groups based on TIDE analysis. **(C)** Distribution of Tumor Immune Dysfunction and Exclusion (TIDE) scores in high and low *PRNP* gene expression groups. **(D)** Differences in immune cell infiltration between high and low *PRNP* gene expression groups. **(E, F)** Heatmaps of immune cell correlation network connections in high and low *PRNP* gene expression groups. (*p < 0.05, **p < 0.01, ***p < 0.001).

Drug sensitivity analysis using the oncoPredict algorithm revealed that patients with high *PRNP* expression exhibited significant resistance to gemcitabine (**Figure 3A**). Furthermore, we assessed the correlation between *PRNP* expression and TIDE, a predictor of immunotherapy response. The High group demonstrated a significantly elevated TIDE score compared to the Low group. Consequently, the proportion of patients predicted as non-responders to immunotherapy was substantially higher in the High group (80.3%) than in the Low group (54.2%). These TIDE results suggest that high *PRNP* expression promotes an immunosuppressive tumor microenvironment conducive to immune escape (**Figures 3B, C**).

To further elucidate the association between *PRNP* and the tumor immune microenvironment, we analyzed the composition of infiltrating immune cell populations. Using the xCell algorithm to estimate relative abundance, we found that the High group was significantly enriched for immunosuppressive cells, including Th2 cells, iDC, B cells, Tregs, and M2 macrophages. Conversely, the Low group exhibited higher scores for immune-active populations such as CD4+Tcm, NKT cells, and Th1 cells. These results indicate that high *PRNP* expression is associated with an immunosuppressive microenvironment, whereas low *PRNP* expression correlates with a profile suggestive of cellular immunity and immune activation (**Figure 3D**).

To systematically investigate the regulatory role of *PRNP* in the tumor immune microenvironment, we constructed correlation heatmaps of immune cell interactions. Analysis revealed a more complex and enhanced cooperative network among various immune cells in the High group compared to the Low group. Critically, a strong and concentrated positive correlation was observed between immunosuppressive cells, notably Tregs and M2 macrophages, suggesting a coordinated immunosuppressive network under high *PRNP* conditions. Concurrently, association analysis between the gemcitabine resistance score and the immune microenvironment revealed distinct infiltration patterns. In the low-score group, the resistance score correlated positively only with Th2 cells, indicating a limited immune shift in early resistance. In stark contrast, the high-score group showed significant negative correlations with cytotoxic/effector populations like NKT cells, CD4+Tem, and CD8+ naive T cells. This aligns with our xCell findings, jointly indicating that gemcitabine resistance is associated with a loss of cytotoxic potential and impaired recruitment of immune cell reserves.

Furthermore, correlating the TIDE score with immune cell infiltration elucidated the immunological basis of its predictive power. In the Low group, the score positively correlated with Monocytes, CD8+ naive T, Pro-B, and CD4+ T cells, but negatively with CD4+ Tem, suggesting initial immune recruitment alongside early effector cell suppression. Conversely, the High group showed positive correlations with B cells, Pro-B cells, and CD4+ naive T cells, while maintaining a strong negative correlation with CD4+ Tem. This pattern reflects a profoundly dysregulated microenvironment, explaining from a cellular perspective why a high TIDE score predicts poor immunotherapy response and robust immune escape (**Figures 3E, F**).

### 3.3 Gemcitabine promotes the production of fibroblastic subsets in pancreatic cancer tumors, which is related to *PRNP* gene expression

**Figure 4:**
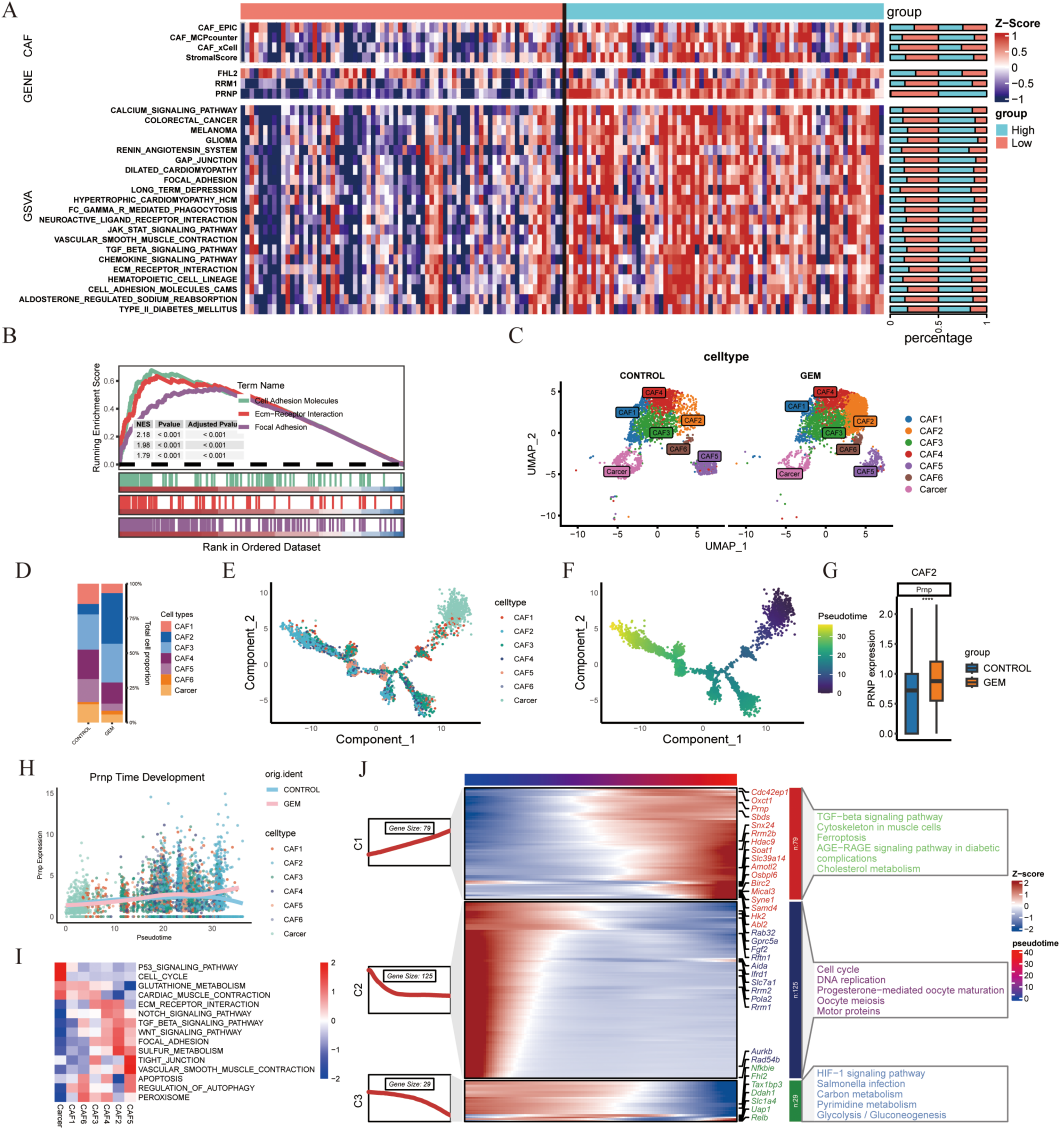
scRNA-seq analysis of gemcitabine-treated mouse pancreatic cancer models **(A)** Heatmap of *PRNP* gene expression in TCGA patients stratified into high and low expression groups. **(B)** GSEA pathway enrichment analysis based on *PRNP* gene expression. **(C)** UMAP plot of CAFs subpopulations in the gemcitabine-treated mouse model from GSE189753. **(D)** Proportional distribution of CAFs subpopulations. E,F. Monocle2 cell trajectory analysis depicting the development of CAFs subpopulations. **(G)** Differential expression of the *Prnp* gene in the CAF2 subpopulation. **(H)***Prnp* gene expression promotes the generation of the CAF2 subpopulation. **(I)** GSVA pathway enrichment heatmap of CAF subpopulations. **(J)** Temporal development of genes and KEGG enrichment heatmap. (****p < 0.0001).

To investigate the potential role of *PRNP* in promoting CAF accumulation, we applied four distinct deconvolution algorithms. These analyses consistently revealed a significant positive correlation between *PRNP* expression and CAF abundance, suggesting that *PRNP* may drive the formation of a CAF-rich tumor microenvironment. Additionally, the level of *PRNP* expression influenced the expression profiles of various genes within the genes, notably *RRM1* gene expression was increased in the *PRNP* high group, while *FHL2* gene expression was not significantly correlated. Finally, GSVA and GSEA analyses demonstrated that high PRNP expression significantly increased the expression of genes involved in epithelial–mesenchymal transition related pathways, such as the TGF-β signaling pathway and the ECM receptor interaction pathway (*p* < 0.05). These findings indicate that PRNP may play a pivotal role in promoting EMT in pancreatic cancer patients and is closely associated with drug resistance (**Figures 4A, B**).

In the single-cell model dataset GSE189753, which focused on the impact of gemcitabine on mouse pancreatic cancer, particular emphasis was placed on the expression patterns and regulatory mechanisms of *Prnp*. On the basis of classification criteria drawn from the literature, eight distinct cell clusters were identified and named according to their primary cellular markers: Monocytes/Macrophages, CAFs, T cells, B cells, Neutrophils, Dendritic cells, Mast cells, and Cancer cells. (**Supplementary Figure S1**) Notably, *Prnp* gene expression was predominantly observed in the CAF subpopulation, with statistical significance (*p* < 0.05). Further subdivision of the CAFs subpopulation revealed that gemcitabine treatment led to an increase in the proportion of CAF2 cell subpopulations in the mouse model (**Figures 4C, D**). CAF2 cells are intimately associated with gemcitabine-induced fibrosis and fall within the inflammatory CAF (iCAF) subtype.

To explore the temporal dynamics of *Prnp* expression during CAF development, we performed cell trajectory analysis using Monocle2. The results indicated that Prnp facilitated the generation of CAF2 subpopulations (**Figures 4E–H**). KEGG pathway enrichment analysis showed that gemcitabine not only promotes the formation of CAF2 subpopulations but also induces EMT and upregulates pathways associated with ferroptosis resistance (**Figures 4I, J**). Collectively, these findings suggest that gemcitabine promotes the generation of pancreatic cancer-associated fibroblast subpopulations, particularly CAF2, which are closely correlated with *Prnp* expression.

### 3.4 Analysis of spatial transcriptome and chemotherapy single-cell population data

**Figure 5:**
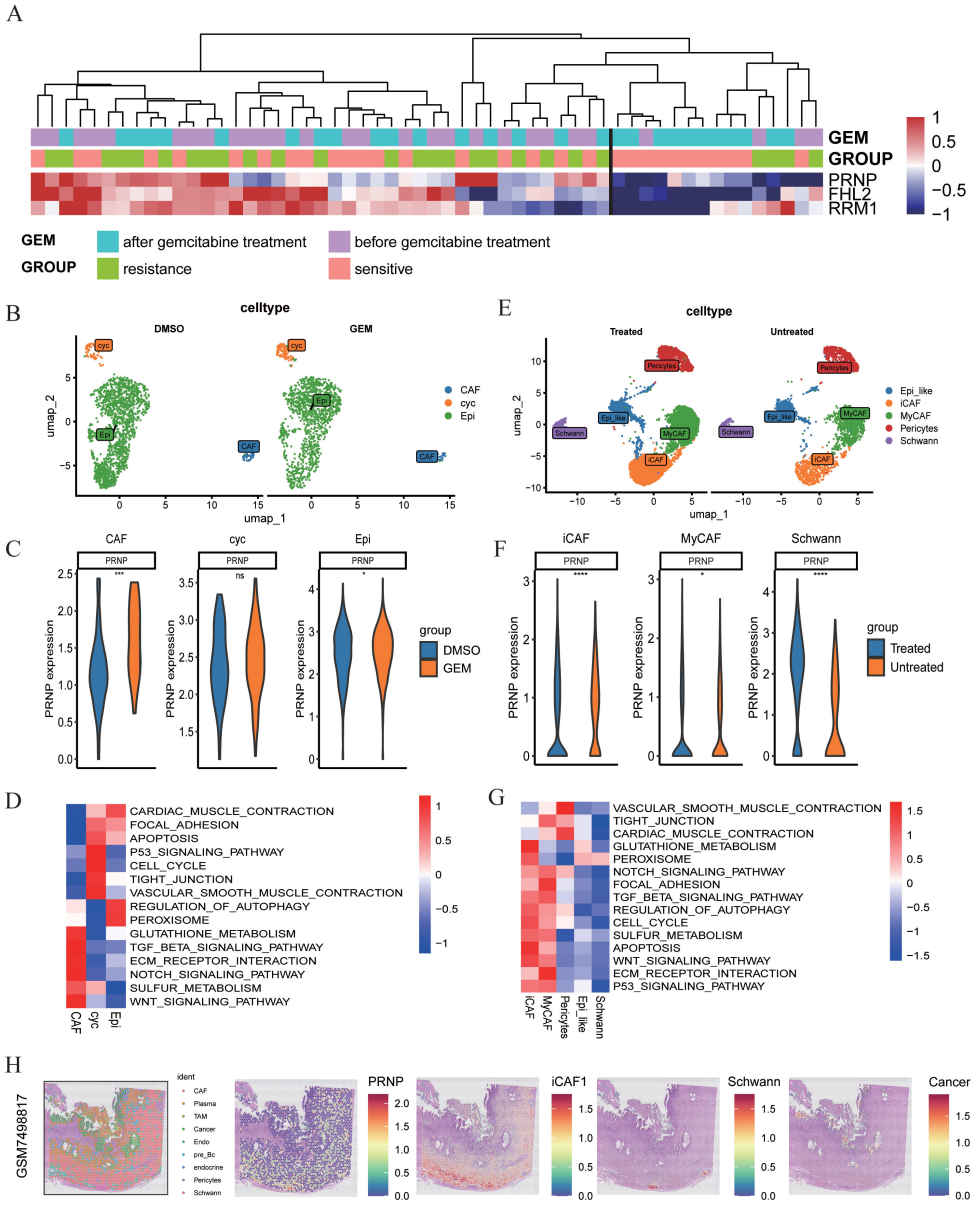
Single-cell and spatial transcriptome analysis of chemotherapy-treated populations **(A)** Expression of 3 genes in the PDX model. **(B)** UMAP plot of pancreatic cancer tissues post-GEM chemotherapy. **(C)** Differential expression of the *PRNP* gene across subpopulations. **(D)** GSVA pathway enrichment heatmap of the subpopulations. **(E)** UMAP plot of CAFs subpopulations in chemotherapy-treated populations. **(F)** Differential expression of the *PRNP* gene in CAF-related subpopulations. **(G)** GSVA pathway enrichment heatmap of CAFs subpopulations. **(H)** Relationships between *PRNP* expression and cell populations in the spatial transcriptome. (*p < 0.05, ***p < 0.001, ****p < 0.0001).

To validate *PRNP* expression in chemotherapy-treated patients, we examined genes expression within the PRJNA694728 gemcitabine-resistant pancreatic cancer patient-derived xenograft (PDX) model transcriptome dataset. The results demonstrated that the genes presented elevated expression in resistant samples following gemcitabine treatment. Notably, *PRNP* expression increased in some sensitive patients after chemotherapy (**Figure 5A**).

To further explore *PRNP* expression during chemotherapy, we analyzed the GSE205013 scRNA-seq dataset of chemotherapy-treated patients (**Supplementary Figures S2C–E**), which included samples from 27 patients. Among them, 7 patients had undergone chemotherapy (4 on the FOLFIRINOX regimen and 3 on the gemcitabine/Abraxane regimen) prior to tissue collection, while the remaining 20 patients were untreated at the time of specimen collection. By isolating CAF subpopulations from single-cell data, we confirmed that the *PRNP* was highly expressed in both iCAFs and Schwann cells post-chemotherapy (*p* < 0.05). Additionally, GSVA analysis revealed significant upregulation of EMT-related pathways in iCAF subpopulations, further confirming that the *PRNP* promotes EMT in chemotherapy-treated populations. Additionally, *PRNP* expression was increased in Schwann cells after chemotherapy (**Figures 5E–G**). Similarly, in the gemcitabine chemotherapy GSE222952 dataset (**Supplementary Figures S2A, B**), *PRNP* expression tended to increase in CAF subpopulations after GEM treatment (**Figures 5B–D**).

In summary, *PRNP* gene expression is elevated in CAF subpopulations of pancreatic cancer patients post-chemotherapy, fostering the emergence of EMT-related genes and drug resistance mechanisms. Based on previous findings, we confirmed that gemcitabine-induced *PRNP* upregulation facilitates the generation of iCAFs in pancreatic cancer. To delve deeper into the expression patterns of *PRNP* in pancreatic cancer samples and the primary cell types that impact them, we conducted an analysis utilizing spatial transcriptomics technology. By integrating data from seven samples, we assessed cell types and identified various populations, including tumor cells, cancer-associated fibroblasts, immune cells, Schwann cells, endothelial cells, and pericytes. Notably, *PRNP* expression was particularly prominent in CAFs, with a strong emphasis on the iCAF subpopulation, as per our previous research. In sample GSM7498817, we observed that iCAFs encircled Schwann cells, suggesting a potential synergistic interaction between these two subpopulations, which may collectively expedite the progression of pancreatic ductal adenocarcinoma (**Figure 5H**, **Supplementary Figure S3**).

### 3.5 Silencing *PRNP* increases pancreatic cancer cell sensitivity to gemcitabine

**Figure 6:**
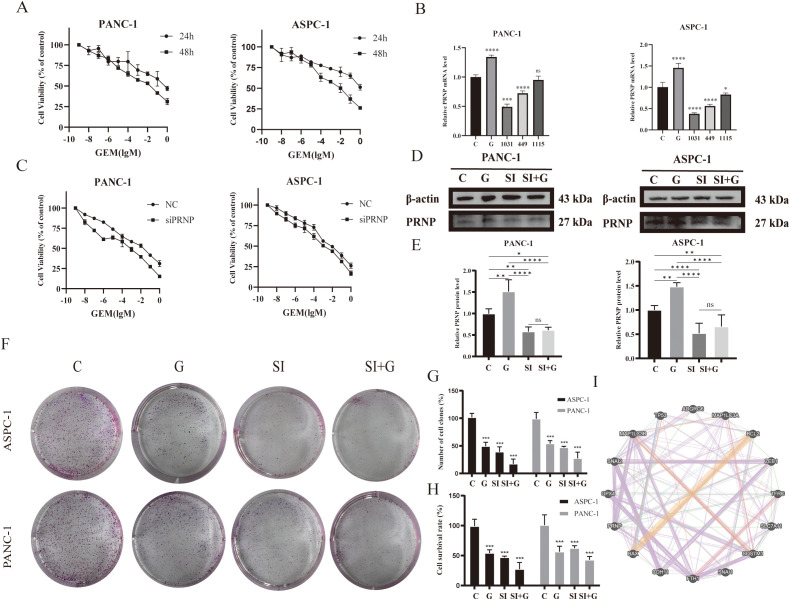
Effects of GEM and *PRNP* expression on cell proliferation **(A)** MTT assay to assess the survival rate of pancreatic cancer cell lines treated with GEM. **(B)** mRNA expression levels of the *PRNP* gene in pancreatic cancer cells. **(C)** MTT assay to assess the survival rate of pancreatic cancer cells with *PRNP* knockdown with GEM treated. **(D, E)** Western blot analysis was used to detect the protein expression of PRNP. **(F, G)** A colony formation assay was used to evaluate the proliferation of pancreatic cancer cells. **(H)** Trypan blue staining was used to detect the proliferation of pancreatic cancer cells. **(I)** Interaction network between the *PRNP* gene and pathway targets. (*p < 0.05, **p < 0.01, ***p < 0.001, ****p < 0.0001).

Bioinformatics analysis revealed that gemcitabine treatment of pancreatic cancer cells increases *PRNP* expression while inducing EMT. To validate this finding, we conducted *in vitro* cellular experiments. We selected the human pancreatic cancer cell lines ASPC-1 and PANC-1 as our study subjects. The MTT method was used to evaluate the impact of gemcitabine on the survival rates of these two pancreatic cancer cell lines (**Figure 6A**). Pancreatic cancer cells were exposed to different concentrations of gemcitabine for varying durations. Compared to the control group, gemcitabine significantly inhibited cell growth in a concentration- and time-dependent manner. After 48 hours of treatment, the half-maximal inhibitory concentrations (IC50) for PANC-1 and ASPC-1 cells were determined to be 48.79 μM and 8.69 μM, respectively. These IC50 values served as the benchmark for guiding subsequent experimental steps.

qRT–PCR and Western blot were employed to confirm that GEM enhances *PRNP* gene expression in pancreatic cancer cells (*p* < 0.05) (**Figures 6D, E**). To delve deeper into the function of *PRNP*, we designed three non-overlapping siRNA sequences targeting *PRNP* using Gemma. Through qRT–PCR screening, si1031 emerged as the most effective siRNA for knocking down *PRNP* in ASPC-1 and PANC-1 cells (*p* < 0.05). Thus, si1031 was used to establish *PRNP* silenced ASPC-1 and PANC-1 cell lines for subsequent experiments (**Figure 6B**).

ASPC-1 and PANC-1 cells were categorized into a negative control group (NC) and a *PRNP* gene knockdown group (siPRNP). After these two groups were exposed to gemcitabine for 48 hours, the results indicated that, at an equivalent drug concentration, the survival rate of the cells in the siPRNP group was markedly lower than that in the NC group. Additionally, the half-maximal inhibitory concentration values for PANC-1 and ASPC-1 cells in the siPRNP group were 27.69 μM and 0.79 μM, respectively, which were significantly lower than those of cells without *PRNP* knockdown (**Figure 6C**).

Cell colony formation and trypan blue staining experiments were conducted, and the silencing of *PRNP* enhanced the toxic effect of gemcitabine on pancreatic cancer cells (**Figures 6F–H**). To further explore the relationships between the *PRNP* and biological processes such as cell apoptosis, migration, invasion, autophagy, and ferroptosis, we selected a series of commonly related markers, including P53, BAX, and BCL2 (related to cell apoptosis); GPX4 and SCL7A11 (related to ferroptosis); and Snail and Slug (related to migration and invasion). Construction of a gene interaction network using GeneMANIA revealed direct associations between *PRNP* and these markers (**Figure 6I**), suggesting that *PRNP* may directly regulate the occurrence and development of these biological pathways.

### 3.6 Knockdown of *PRNP* increases the inhibitory effect of gemcitabine on pancreatic cancer cell migration and invasion

**Figure 7:**
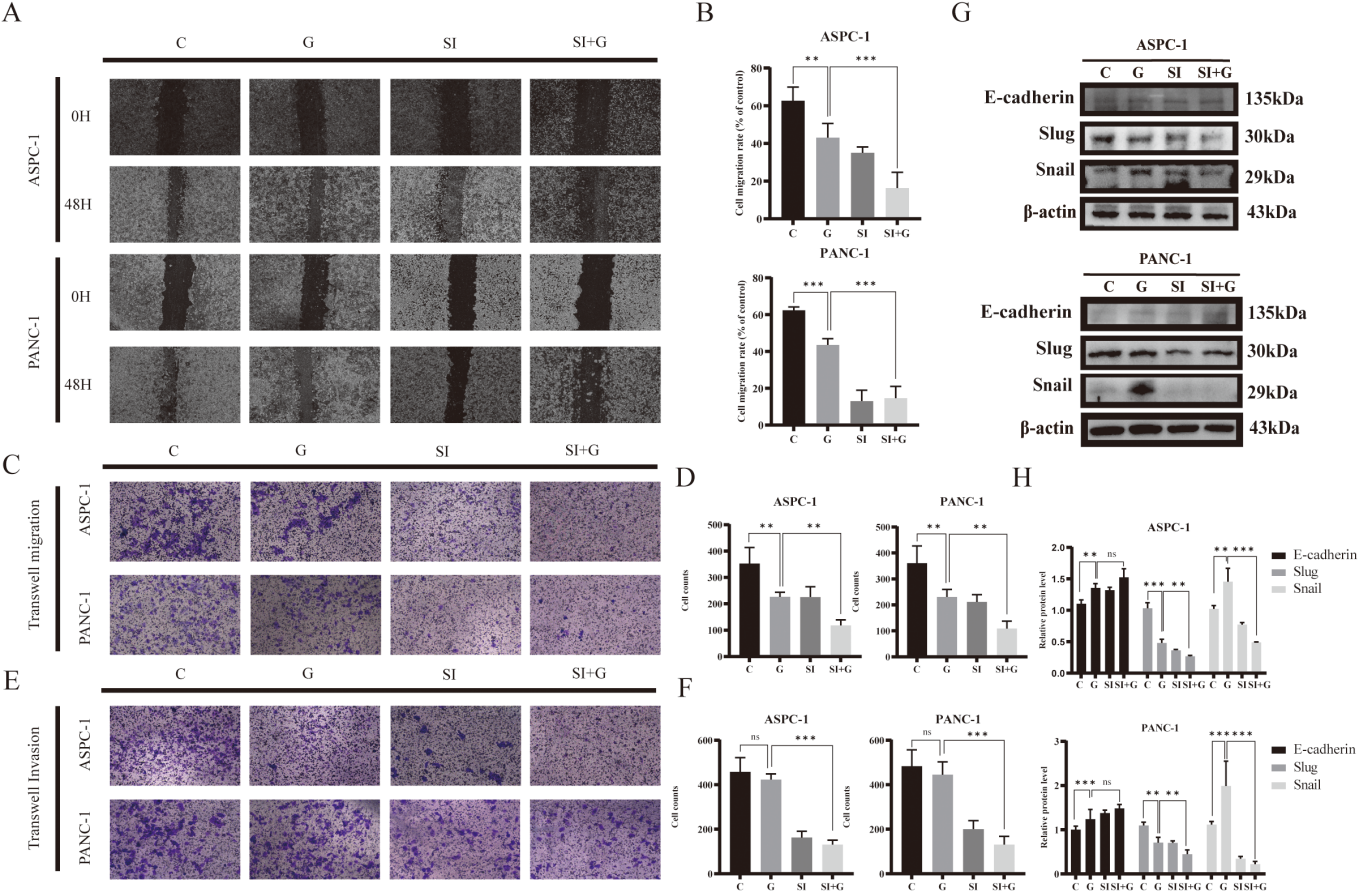
Effects of GEM and *PRNP* expression on cell migration and invasion **(A, B)** Wound-healing assay to detect the migration of pancreatic cancer cells (×100 magnification). **(C, D)** Transwell migration assays were used to assess cell migration ability (×100 magnification). **(E, F)** Transwell invasion assays were used to evaluate cell invasion ability (×100 magnification). **(G, H)** Western blot analysis was used to detect the expression of EMT related proteins. (**p < 0.01, ***p < 0.001).

A wound-healing assay was performed to evaluate cell migration ability. Compared with those in the control group and the GEM group, the cell migration rates in both the siPRNP and the siPRNP+GEM groups gradually decreased (**Figures 7A, B**). Furthermore, the cell migration ability of the siPRNP+GEM group was notably lower than that of the GEM group (*p* < 0.05). These findings indicate that the knockdown of *PRNP* can potentiate the inhibitory effect of gemcitabine on the migration of pancreatic cancer cells.

Transwell migration assay to confirm alterations in cell migration ability, we discovered that the number of cells that crossed the Transwell chamber in the GEM group was markedly lower than that in the control group, indicating the inhibitory effect of gemcitabine on pancreatic cancer cell migration. Furthermore, the number of cells that passed through the chamber in the siPRNP+GEM group was even greater than that in the GEM group, suggesting that *PRNP* knockdown augments the inhibitory effect of gemcitabine on the migration of human pancreatic cancer cells (*p* < 0.05). The results of the Transwell invasion assay aligned with those of the migration assay (*p* < 0.05), demonstrating that silencing *PRNP* enhances the inhibitory effect of gemcitabine on pancreatic cancer cell invasion (**Figures 7C–F**).

After 48 hours of gemcitabine treatment, we observed an increasing trend in the protein expression of E-cadherin, whereas the protein expression of Snail was increased and the protein expression of Slug was decreased (*p* < 0.05). After the *PRNP* gene was silenced, the promotion of E-cadherin protein expression by GEM and the decrease in the protein expression of Snail and Slug were significantly increased (*p* < 0.05) (**Figures 7G, H**). Taken together, the results of the wound-healing and Transwell assays indicate that GEM affects the EMT pathway to inhibit the migration and invasion abilities of pancreatic cancer cells and that silencing the *PRNP* gene synergistically inhibits the EMT process in pancreatic cancer cells with GEM.

### 3.7 *PRNP* regulates ferroptosis and sensitizes pancreatic cancer cells to gemcitabine

**Figure 8:**
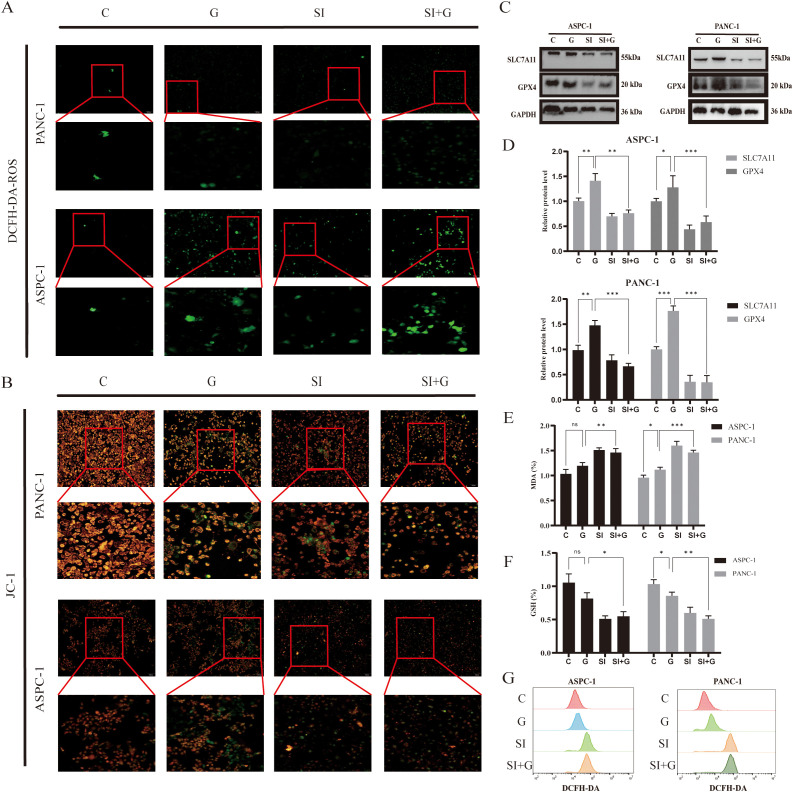
Relationship between GEM and *PRNP* expression ferroptosis **(A)** DCFH-DA fluorescence detection of reactive oxygen species (ROS) changes in cells (×100 magnification). **(B)** JC-1 fluorescence detection of changes in the mitochondrial membrane potential of cells (×100 magnification). **(C, D)** Western blot analysis of the expression of ferroptosis-related proteins. **(E)** Changes in the MDA content. **(F)** Changes in the GSH content. **(G)** Flow cytometry detection of reactive oxygen species (ROS) changes in cells. (*p < 0.05, **p < 0.01, ***p < 0.001).

Ferroptosis, a form of programmed cell death reliant on iron and ROS, plays a crucial role in tumorigenesis and progression and is tightly linked to drug resistance in cancer. To explore the role of *PRNP* in ferroptosis, we employed the DCFH-DA ROS fluorescent probe to monitor ROS production in pancreatic cancer cells after GEM treatment. Upon silencing the *PRNP* gene, GEM markedly augmented ROS generation in pancreatic cancer cells (**Figure 8A**).

The JC-1 fluorescent probe is a key indicator of ferroptosis, as ferroptosis is associated with specific changes in mitochondrial membrane potential. A decrease in mitochondrial membrane potential causes the JC-1 probe to shift from red to green fluorescence. We observed that, upon silencing the *PRNP* gene, GEM significantly accelerated the reduction in the mitochondrial membrane potential in pancreatic cancer cells (**Figure 8B**), suggesting that silencing *PRNP* facilitates ferroptosis induction.

From a molecular standpoint, upon the introduction of GEM, the expression levels of the key ferroptosis-related proteins SLC7A11 and GPX4 increase, conferring resistance to ferroptosis. However, when the *PRNP* was silenced, the expression of these two proteins was suppressed, thereby promoting the induction of ferroptosis (*p* < 0.05) (**Figures 8C, D**). Further exploration of alterations in the content of glutathione (GSH) and malondialdehyde (MDA), revealed that *PRNP* gene silencing led to significant depletion of intracellular GSH and MDA levels rise (*p* < 0.05). With GSH depletion and MDA rise, cells become more susceptible to ROS-induced apoptosis (**Figures 8E, F**). Flow cytometry-based ROS detection further confirmed a marked increase in intracellular ROS levels following *PRNP* silencing (**Figure 8G**). These findings indicate that *PRNP* silencing enhances gemcitabine-induced ferroptosis in pancreatic cancer cells.

To determine whether *PRNP* deficiency-induced ferroptosis activation specifically relies on the downregulation of SLC7A11 and GPX4 protein levels, this study conducted ferroptosis inhibitor rescue experiments in *PRNP* knockdown pancreatic cancer cells.

**Figure 9:**
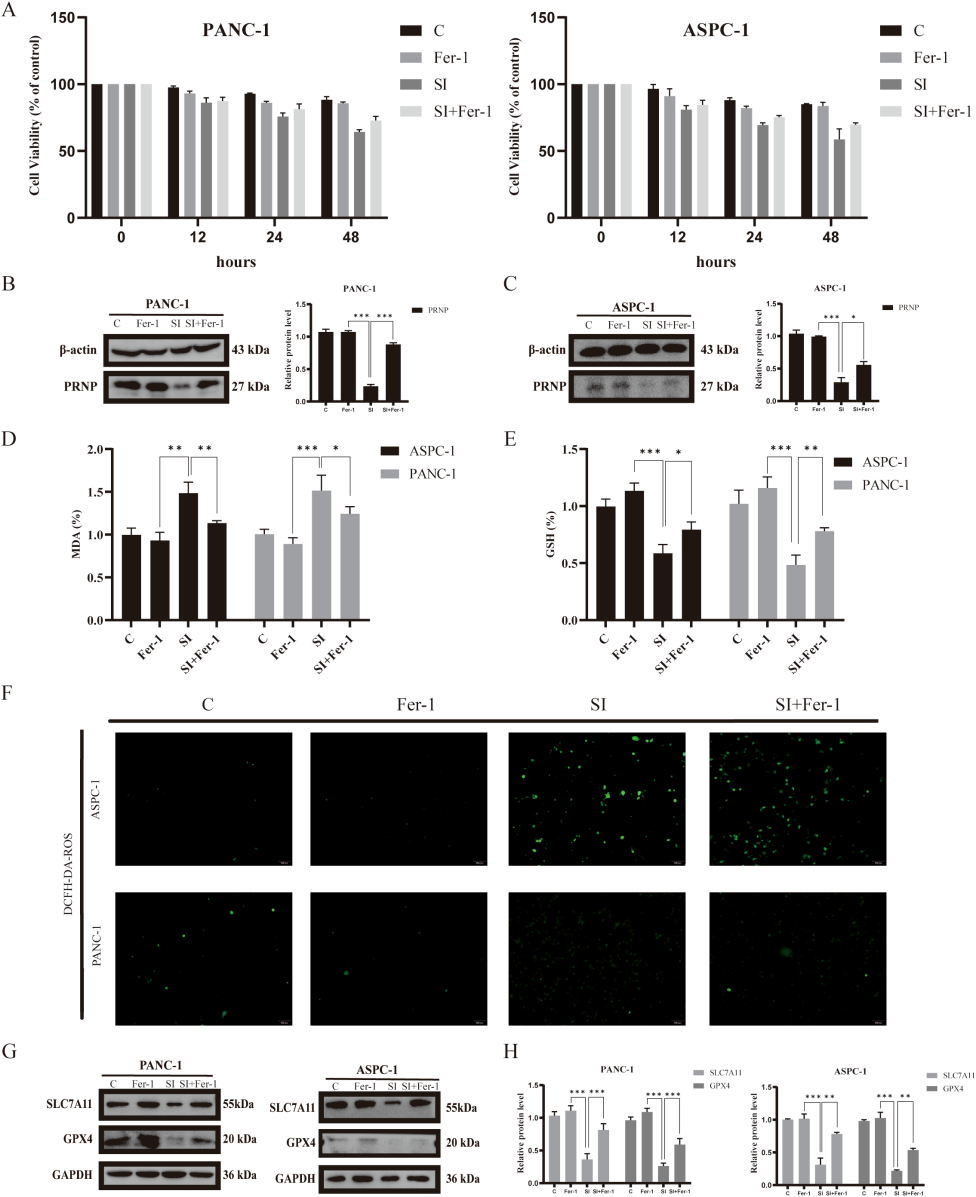
Relationship between Ferrostatin-1 and *PRNP* expression and ferroptosis **(A)**. MTT assay to assess the survival rate of pancreatic cancer cell lines treated with Ferrostatin-1. **(B, C)** Western blot analysis of the expression of PRNP proteins. **(D)** Changes in the MDA content. **(E)** Changes in the GSH content. **(F)** DCFH-DA fluorescence detection of reactive oxygen species (ROS) changes in cells (×100 magnification). **(G, H)** Western blot analysis of ferroptosis-related protein expression after Ferrostatin-1 and siPRNP treatment. (*p < 0.05, **p < 0.01, ***p < 0.001).

MTT analysis demonstrated that adding 1 μM Ferrostatin-1 to pancreatic cancer cells with silenced *PRNP* gene significantly suppressed the cell death process, initially suggesting an intrinsic link between Ferrostatin-1 and *PRNP* gene expression regulation (**Figure 9A**). Western blot analysis further revealed that, compared to *PRNP* silenced pancreatic cancer cells, the PRNP protein expression level in Ferrostatin-1 - treated silenced cells exhibited an upward trend, providing robust protein level evidence for the relationship between Ferrostatin-1 and *PRNP* gene expression (**Figures 9B, C**).

To explore whether Ferrostatin-1 and *PRNP* in silenced pancreatic cancer cells are involved in the ferroptosis pathway, we assessed key ferroptosis related indicators. The results showed that after Ferrostatin-1 treatment, intracellular GSH levels significantly increased, indicating enhanced cellular antioxidant capacity and alleviated oxidative stress - induced damage. Concurrently, MDA levels were markedly inhibited, suggesting reduced intracellular lipid peroxidation and blocked lipid peroxidation chain reactions (*p* < 0.05) (**Figures 9D, E**). Additionally, ROS fluorescent probe detection showed a decrease in intracellular ROS fluorescence intensity, indicating reduced ROS levels (**Figure 9F**). Collectively, these findings imply that Ferrostatin-1 may inhibit ferroptosis by regulating cellular redox balance, and this process may be associated with *PRNP*.

Simultaneously, depth exploration was conducted into the molecular mechanism underlying ferroptosis. Western blot analysis that treatment with ferroptosis inhibitors exerted a significant reversal effect on the down - regulation of SLC7A11 and GPX4 protein expression induced by *PRNP* knockdown (**Figures 9G, H**). Specifically, following the administration of ferroptosis inhibitors, the previously diminished levels of SLC7A11 and GPX4 proteins, which had been suppressed due to *PRNP* silencing, were restored to a notable extent. This finding provides crucial insights into the interplay between *PRNP*, ferroptosis inhibitors, and the key ferroptosis - related proteins SLC7A11 and GPX4.

Integrating the results of *PRNP* silencing and pharmacological interventions, we conclude that *PRNP* exerts a critical ferroptosis-suppressing role in pancreatic cancer cells by positively regulating SLC7A11 and GPX4 protein expression. Notably, *PRNP* silenced pancreatic cancer cells exhibit significantly enhanced ferroptosis responses when exposed to gemcitabine, suggesting that *PRNP* dysfunction may sensitize pancreatic cancer cells to gemcitabine-induced ferroptosis.

### 3.8 Effects of gemcitabine on apoptosis and autophagy in *PRNP*-silenced pancreatic cancer cells

**Figure 10:**
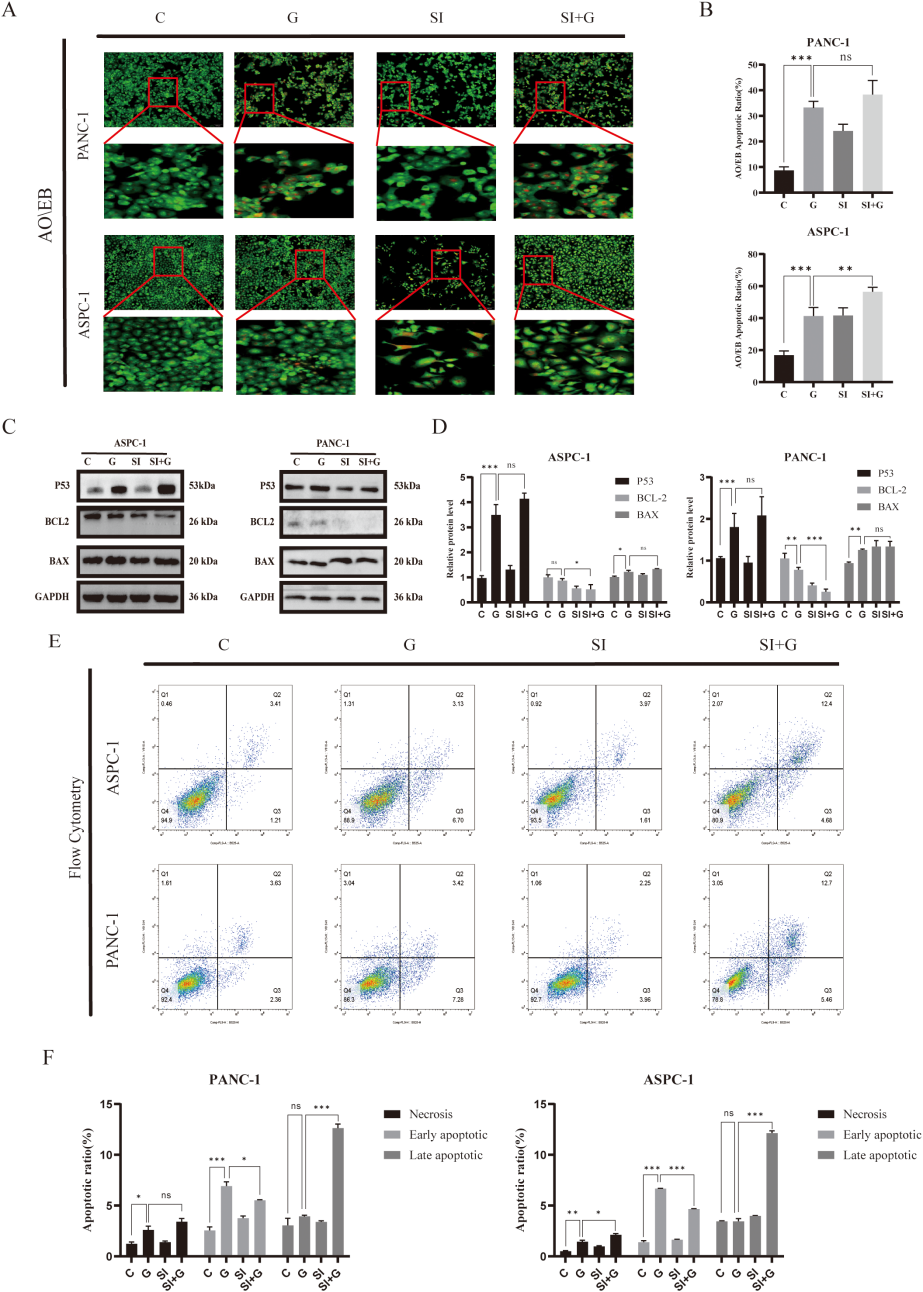
Relationship between *PRNP* expression, cellular autophagy, and apoptosis **(A, B)**. AO/EB fluorescence detection of changes in cellular apoptosis (×100 magnification). **(C, D)** Western blot analysis of the expression of apoptosis-related proteins. **(E, F)**. Flow cytometry detection of changes in the proportion of apoptotic cells. (*p < 0.05, **p < 0.01, ***p < 0.001).

To investigate the synergistic apoptotic effect of gemcitabine and *PRNP* silencing in pancreatic cancer cells, we first used MTT assays and colony formation analysis to quantify cell viability and clonogenic survival. Subsequent AO/EB fluorescence staining revealed that gemcitabine alone induces apoptosis, and *PRNP* silencing also exerts a pro-apoptotic effect. Importantly, the combination of gemcitabine and siPRNP produced a synergistic apoptotic response, indicating that *PRNP* silencing enhances gemcitabine-induced apoptosis in pancreatic cancer cells (**Figures 10A, B**).

Perform Western blot analysis to evaluate the expression levels of key apoptosis regulatory factors P53, BCL2, and BAX. In pancreatic cancer cells treated with GEM, we observed that compared with the control group, P53 and BAX protein expressions were significantly up-regulated, while BCL2 protein levels were down regulated (*p* < 0.05). When *PRNP* is silenced alone, BCL2 protein levels are downregulated (*p* < 0.05), while increasing BAX levels does not affect TP53. It is worth noting that compared with the single treatment group, the combination therapy of siPRNP and GEM showed a synergistic effect, leading to an increase in P53 and BAX expression, while BCL2 protein expression was inhibited (*p* < 0.05). These protein expression patterns collectively indicate that *PRNP* silencing enhances GEM induced cell apoptosis (**Figures 10C, D**).

Finally, flow cytometry-based apoptosis detection revealed a significant increase in the proportion of apoptotic cells in the siPRNP+GEM group (*p* < 0.05), further confirming that the combination of GEM and *PRNP* silencing promoted apoptosis (**Figures 10E, F**).

## 4 Discussion

Gemcitabine has long been a crucial and widely used drug in the treatment of PDAC (18). Despite the ability of chemotherapy regimens to improve survival rates in both first-line and second-line settings, most patients eventually develop drug resistance, leading to tumor recurrence and maintaining long-term survival rates at a relatively low level (19, 20). Currently, the molecular mechanisms underlying gemcitabine resistance phenotypes remain unclear. In this study, we utilized a comprehensive multi-omics integrated analysis approach to explore potential targets of gemcitabine resistance mechanisms in pancreatic cancer (21). By examining the alterations in cellular differential gene expression when gemcitabine interacts with cancer cells, we pinpointed a group of genes that are consistently and stably overexpressed. Notably, irrespective of the cell line in which gemcitabine is targeted or the presence of drug-resistant phenotypes, this gene set demonstrates robust expression patterns, encompassing well-known key gemcitabine resistance genes such as *RRM1* and *RRM2* (22, 23).

Given potential discrepancies between protein levels and gene expression, we performed an exhaustive proteomic analysis to investigate the specific effects of gemcitabine on the proteins encoded by pertinent genes in pancreatic cancer cells. Our findings revealed that the expression patterns of these proteins play diverse roles in gemcitabine resistance mechanisms. Notably, the *PRNP* gene, which is characterized by persistent resistance, emerged as the focal point of our research endeavors (24, 25). Enrichment analysis confirmed that *PRNP* is linked to drug resistance mechanisms, including EMT and ferroptosis (26, 27). Previous studies have demonstrated that *PRNP* plays a crucial role in regulating tumor growth and differentiation and enhances resistance to traditional therapeutic approaches (28–30).

Pancreatic cancer exhibits inherent resistance to conventional therapies, largely attributable to its pronounced tumor heterogeneity, highly desmoplastic stroma, and profoundly immunosuppressive tumor microenvironment (31, 32). Supporting the role of *PRNP* within this context, existing evidence indicates that its expression is associated with various immune cells, including T cells and DCs, and shows a direct correlation with the immunosuppressive gene *IDO1* (33, 34). Further reinforcing its immunomodulatory function, *PRNP* expression in glioma cell lines has been linked to responses to IFN-α, underscoring its potential involvement in immune microenvironment regulation. In pancreatic cancer specifically, elevated *PRNP* expression is recognized for its role in activating EMT-related pathways, thereby promoting tumor invasion and conferring resistance to chemotherapy (35–37). Gemcitabine stimulates the upregulation of *PRNP*, which subsequently promotes the emergence of iCAF subpopulations and accelerates the EMT process, thereby potentiating the development of drug-resistant tumor phenotypes (38). Spatial transcriptome and single-cell data from chemotherapy-treated patients further indicate that high *PRNP* expression in clinical chemotherapy patients accelerates the EMT process in iCAFs and influences the subpopulations of both iCAFs and Schwann cells (39, 40). However, due to inherent limitations of scRNA-seq technology, research on Schwann cells in PDAC remains limited.

Schwann cells, the predominant glial cell population in the peripheral nervous system, have been implicated in promoting tumor progression and poor prognosis in PDAC through reciprocal interactions with cancer-associated fibroblasts (41, 42). Specifically, Schwann cells induce phenotypic conversion of CAF into more aggressive subtypes, including basal-like CAFs and iCAF, via interleukin-1α (IL-1α)-mediated signaling. Emerging evidence suggests that chemotherapy may exacerbate these malignant interactions by upregulating *PRNP* expression, thereby enhancing Schwann cell-iCAF crosstalk at the tumor-stromal interface (43, 44).

Mechanistically, proteolytic cleavage of glycosylphosphatidylinositol (GPI)-anchored *PRNP* generates a soluble isoform that functions as both an autocrine and paracrine neurotrophic mediator. Notably, in PDAC, *PRNP* predominantly exists as pro-PrP rather than its GPI-anchored form, enabling direct interaction with filamin A (FLNa) (45). This binding disrupts FLNa-mediated cytoskeletal remodeling, leading to enhanced PDAC cell proliferation, migration and invasion. Consistent with these findings, combined *PRNP* silencing and GEM treatment significantly suppressed EMT progression *in vitro*, suggesting that *PRNP* serves as a critical regulator of pancreatic cancer aggressiveness through modulation of tumor-neural interactions.

*PRNP* silencing reprograms cellular fate through a dual molecular axis: on the one hand, suppressing EMT pathway progression to induce phenotypic reversal; on the other hand, precisely modulating the core execution network of ferroptosis, resulting in dose-dependent reductions in GPX4 and SLC7A11 protein levels, thereby triggering lipoperoxidative imbalance. Numerous researchers have highlighted the importance of ferroptosis in tumorigenesis and malignant progression. From a bioinformatics perspective, we pinpointed *PRNP* as a gene intricately linked to ferroptosis (46). When gemcitabine is administered for the treatment of pancreatic cancer and leads to high expression, it induces the emergence of ferroptosis resistance mechanisms within tumor cells. Similarly, elevated oxidation of lipids and proteins has been noted in the brains of normal mice subjected to *PRNP* silencing. These observations suggest that the physiological role of the *PRNP* is intimately tied to the cellular antioxidant defense system (47, 48). Studies have demonstrated that *PRNP* modulates the expression of MAPK and FOXO3a via the epidermal growth factor receptor signaling pathway (15), influencing the emergence of platinum resistance in colorectal cancer. Concurrently, the *RBMS1/PRNP* axis enhances oxaliplatin resistance in colon cancer, thereby contributing to ferroptosis resistance (49). These findings suggest that *PRNP* is a crucial gene in the ferroptosis process and a potential therapeutic target for reversing drug resistance in PDAC.

Mechanistic analyses demonstrate that *PRNP* functions as a “molecular gatekeeper” in ferroptosis defense by maintaining redox homeostasis within the SLC7A11/GPX4 axis. When *PRNP* is silenced, the expression levels of GPX4 and SLC7A11, which are essential for ferroptosis, are decreased, confirming that *PRNP* silencing facilitates the induction of ferroptosis, accelerates glutathione depletion, and elevates ROS levels. As a pivotal gene in the ferroptosis pathway, *PRNP* also participates in cell signaling, autophagy, and antiapoptotic mechanisms. Remarkably, *PRNP* silencing demonstrates pathway-specific modulation, selectively disrupting key autophagy-related proteins (LC3, P62) and apoptotic regulators (BCL2, BAX). Excessive or prolonged autophagy can disrupt mitochondrial structure during tumorigenesis and metastasis, ultimately triggering cellular autophagy (50, 51). Additionally, silencing *PRNP* concurrently decreases mitochondrial membrane potential in pancreatic cancer cells, leading to mitochondrial damage and stimulating autophagy (52). Compared with neurons in the mouse hippocampus, which express cellular prion protein, hippocampal neurons deficient in the *PRNP* exhibit increased autophagy. This finding underscores the occurrence of autophagy in neuronal cells subsequent to the downregulation of the *PRNP* (52).

**Figure 11:**
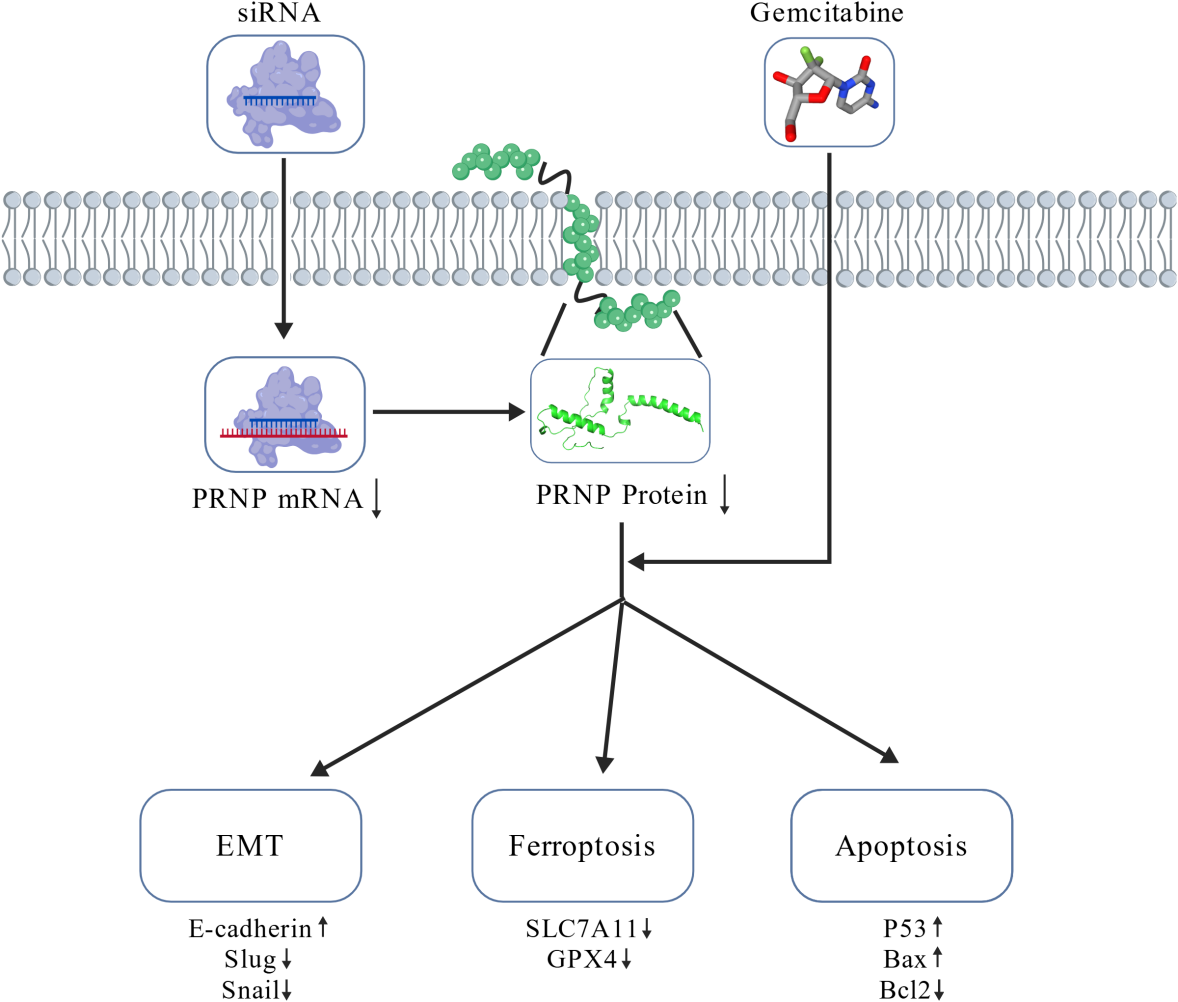
Mechanism diagram.

Gemcitabine exerts cytotoxicity primarily through activation of the canonical TP53 dependent apoptotic pathway, while concurrently exerting dual regulatory roles: suppressing EMT progression and inducing protective autophagy to maintain a dynamic equilibrium between cellular survival and death. Notably, gemcitabine monotherapy suppresses ferroptosis, suggesting that chemotherapeutic agents employ multiple antioxidant defense mechanisms to preserve cellular homeostasis. However, *PRNP* silencing fundamentally alters gemcitabine’s therapeutic profile by disrupting cellular antioxidant defenses through downregulation of SLC7A11 and GPX4, thereby exceeding redox thresholds. The combination regimen induces hallmark ferroptotic phenotypes, characterized by mitochondrial membrane potential depolarization and excessive ROS accumulation. This shift converts the predominant apoptotic cell death mode (observed in monotherapy) into a mixed apoptotic and ferroptosis phenotype with marked enhancement of ferroptosis contributions, resulting in synergistic lethality. Mechanistic validation through apoptotic protein expression analysis (P53, BCL2, BAX) confirmed that *PRNP* depletion potentiates gemcitabine-induced apoptosis in pancreatic cancer cells. Consistent with these findings, elevated *PRNP* expression has been correlated with apoptosis suppression in osteosarcoma, melanoma, colorectal carcinoma, and normal brain tissue (53, 54) (**Figure 11**).

This study systematically delineates the differential regulatory networks governing pancreatic cancer cell death modes induced by *PRNP* silencing, gemcitabine monotherapy, and their combination. For the first time, through integrated multi-cohort analysis and functional cell experiments, it confirms that *PRNP* serves as a potential molecular target for gemcitabine therapy in pancreatic cancer. The research reveals a significant correlation between *PRNP* expression levels and gemcitabine resistance, providing a novel perspective for deciphering the mechanisms of chemotherapy resistance in pancreatic cancer. Furthermore, it elucidates the molecular mechanisms underpinning the synergistic therapeutic effects mediated by multimodal pathway crosstalk. Although this study successfully uncovered key mechanisms of *PRNP* involvement in gemcitabine resistance, offering a theoretical basis for targeted reversal of resistance, and holds significant scientific value and clinical implications for optimizing pancreatic cancer chemotherapy strategies and improving patient prognosis, the following limitations remain: Firstly, the lack of validation in *in vivo* animal models means the biological function of *PRNP* within the tumor microenvironment has not been systematically elucidated at the whole-organism level, representing a critical bottleneck hindering its clinical translation. Secondly, the regulatory network governing *PRNP* expression in specific cell subpopulations such as inflammatory cancer-associated fibroblasts and Schwann cells, along with its precise molecular mechanisms in tumor-stroma interactions, requires further in-depth exploration. Subsequent research must prioritize overcoming these bottlenecks to comprehensively realize the clinical application potential of *PRNP* as a therapeutic target.

## 5 Conclusion

In summary, gemcitabine triggers EMT and ferroptosis resistance mechanisms in pancreatic cancer cells by upregulating *PRNP* expression. By silencing of *PRNP* abrogated GEM-induced EMT and conferred concomitant sensitization to ferroptosis, thereby promoting apoptosis in pancreatic cancer cells. These findings underscore the role of *PRNP* as a marker for both the EMT and ferroptosis pathways. Furthermore, bioinformatics studies revealed that *PRNP* can mediate EMT in iCAF and is associated with Schwann cells under chemotherapy conditions. Collectively, these findings imply that *PRNP* could serve as target for gene therapy in pancreatic cancer, offering new insights for the future clinical application of gemcitabine.

## Data Availability

The dataset used in this study can be found in an online repository. All datasets are publicly available.The name of the repository and the access number can be found in the methodology section of the article.

## Supplementary material

Supplementary Figure 1 scRNA-seq Analysis GSE189753. **(A)** UMAP plot of celltype above GSE189753. **(B)** FeaturePlot *PRNP* gene expression. **(C)** DotPlot celltype markers gene expression.

Supplementary Figure 2 scRNA-seq Analysis GSE222952 and GSE205013. **(A)** GSE222952 FeaturePlot *PRNP* gene expression. **(B)** GSE222952 DotPlot celltype markers gene expression **(C)** UMAP plot of celltype above GSE205013. **(D)** GSE205013 FeaturePlot *PRNP* gene expression. **(E)** GSE205013 DotPlot celltype markers gene expression.

Supplementary Figure 3 scRNA-seq Analysis GSE235452. **(A)** Estimated abundances of celltype above GSE235452. **(B)** GSE235452 DotPlot celltype markers gene expression. **(C)** Estimated abundances of *PRNP*, iCAFs, Schwann and Cancer are plotted for each slide.
