# Supplementary figures and images for: Integrated multi-omics elucidates PRNP knockdown-mediated chemosensitization to gemcitabine in pancreatic ductal adenocarcinoma
Source: Front Immunol. 2025 Nov 27;16:1667835. doi: 10.3389/fimmu.2025.1667835 (PMC12695746; doi:10.3389/fimmu.2025.1667835)

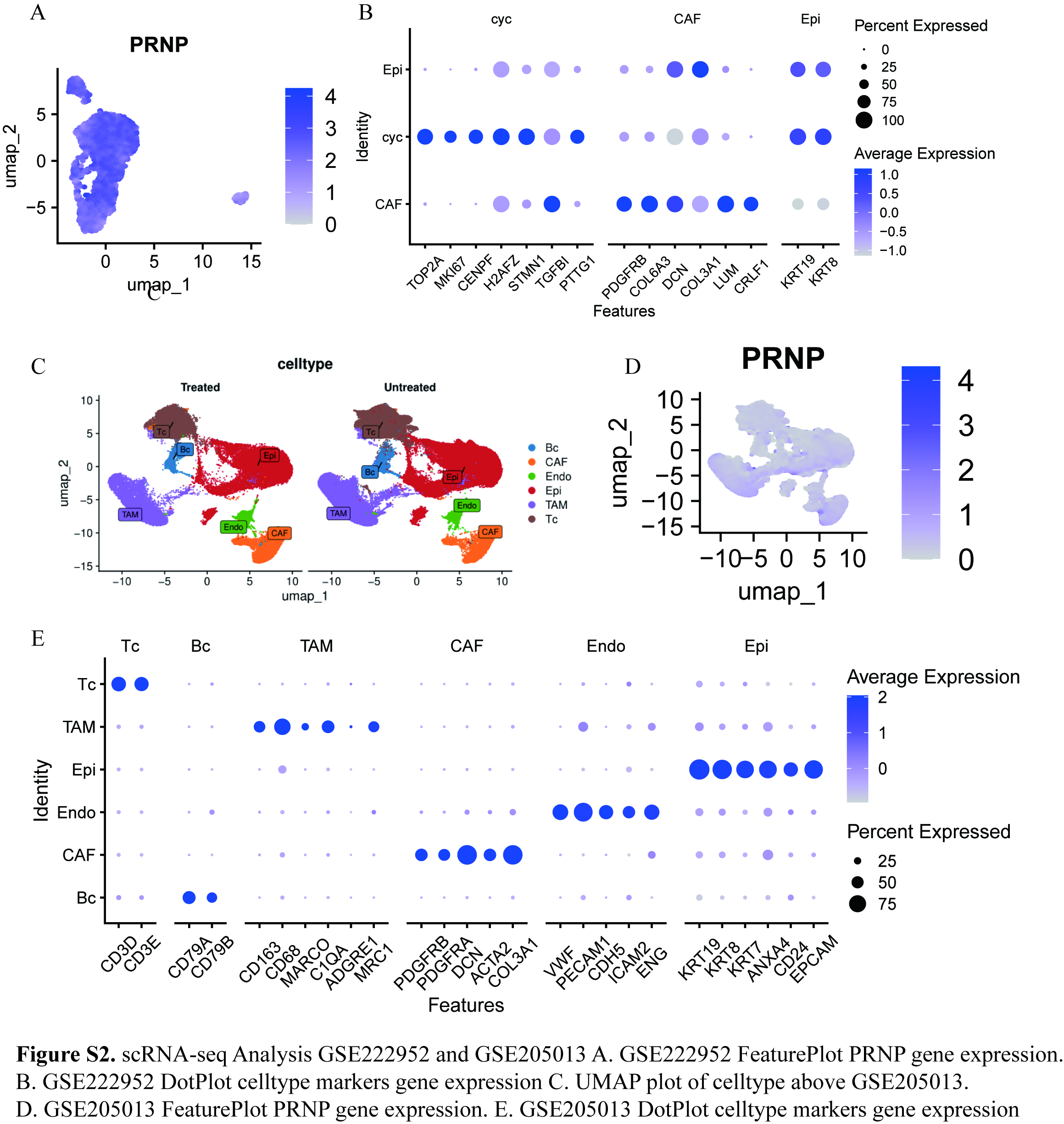

Supplement: Supplementary Figure 1 — scRNA-seq Analysis GSE189753. (A) UMAP plot of celltype above GSE189753. (B) FeaturePlot PRNP gene expression. (C) DotPlot celltype markers gene expression. [file Image1.tif]

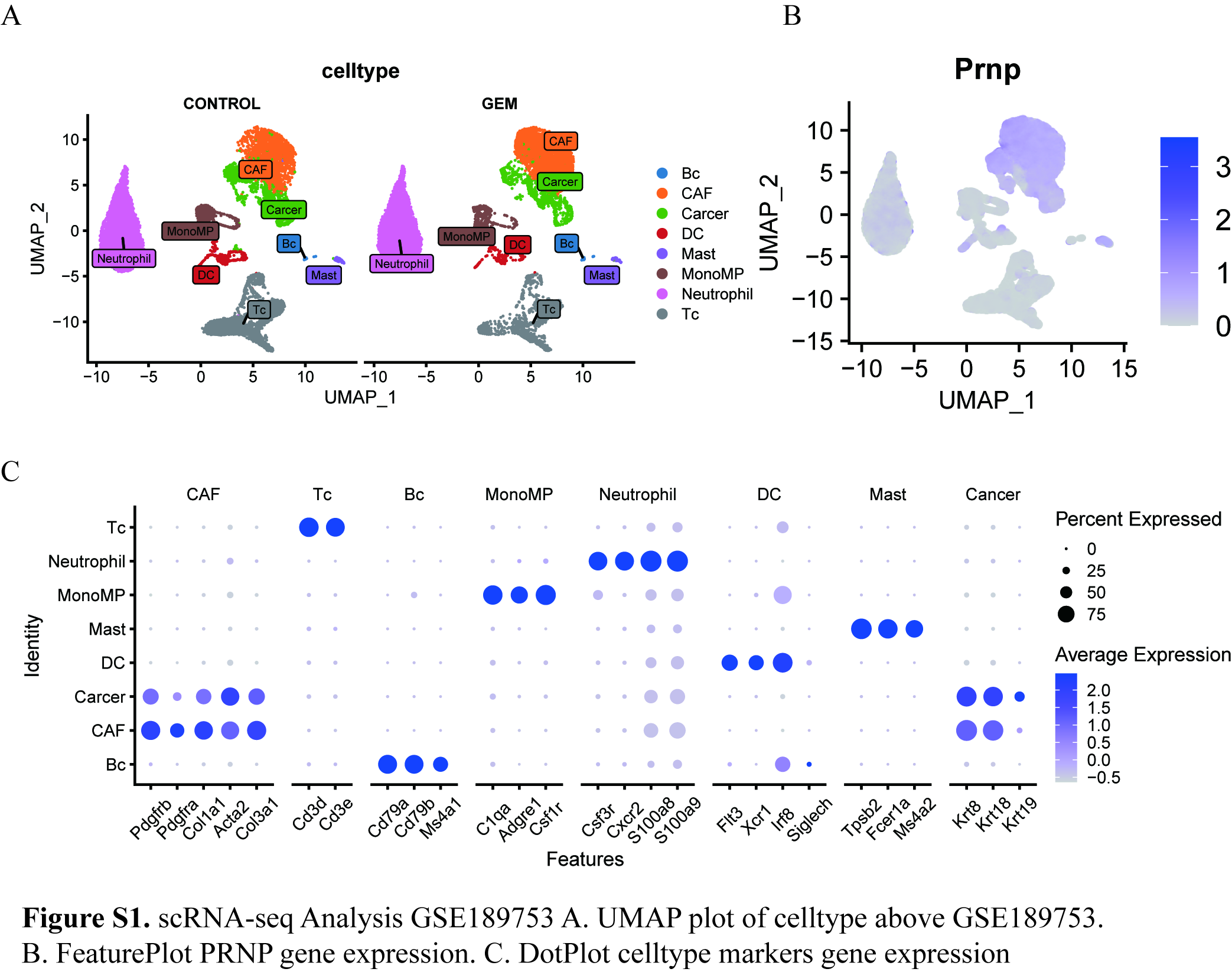

Supplement: Supplementary Figure 2 — scRNA-seq Analysis GSE222952 and GSE205013. (A) GSE222952 FeaturePlot PRNP gene expression. (B) GSE222952 DotPlot celltype markers gene expression (C) UMAP plot of celltype above GSE205013. (D) GSE205013 FeaturePlot PRNP gene expression. (E) GSE205013 DotPlot celltype markers gene expression. [file Image2.tif]

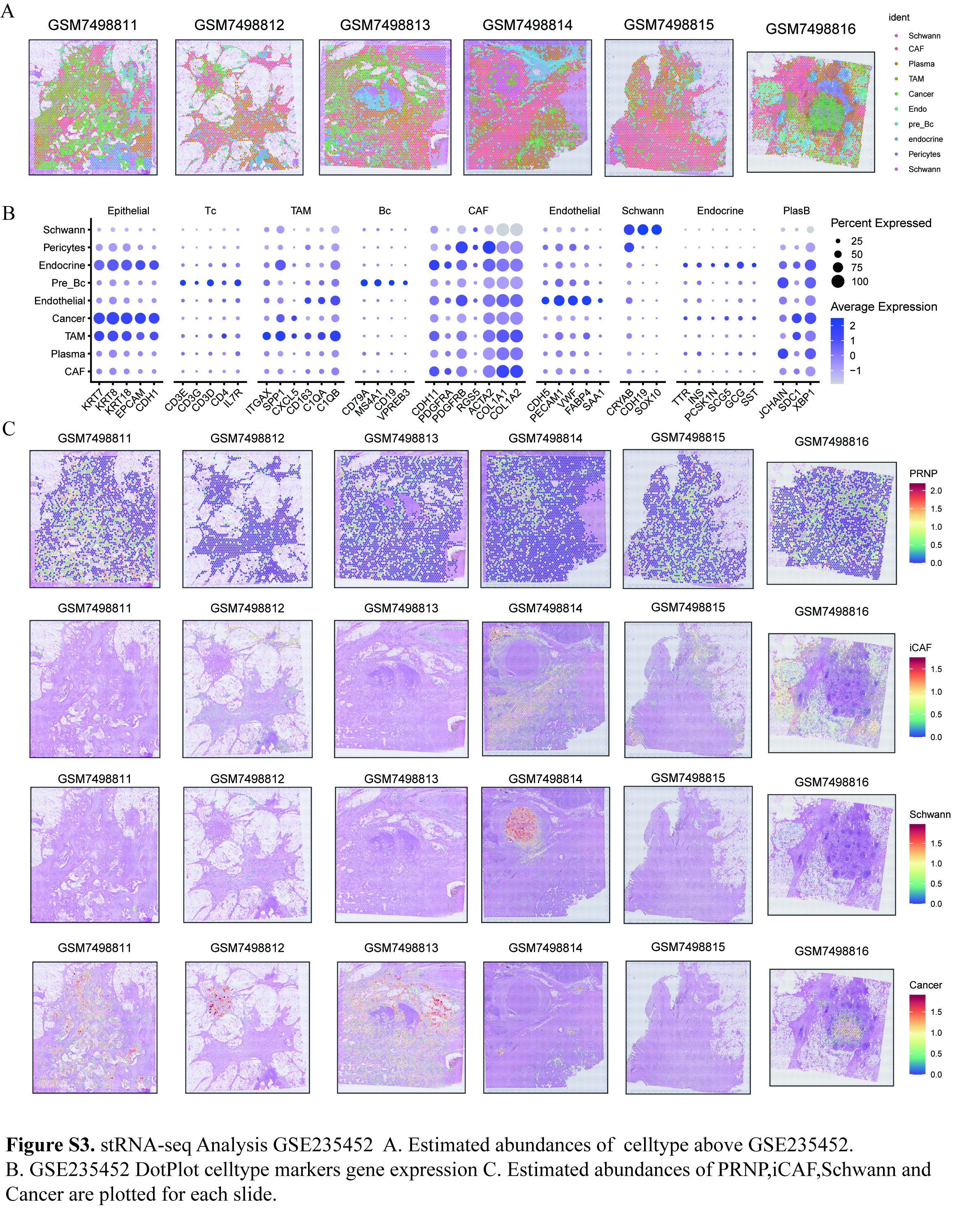

Supplement: Supplementary Figure 3 — scRNA-seq Analysis GSE235452. (A) Estimated abundances of celltype above GSE235452. (B) GSE235452 DotPlot celltype markers gene expression. (C) Estimated abundances of PRNP, iCAFs, Schwann and Cancer are plotted for each slide. [file Image3.tif]
